# Preferences for gene therapy in Duchenne muscular dystrophy: insights from patient and caregiver interviews and attribute development

**DOI:** 10.3389/fphar.2025.1662586

**Published:** 2025-09-29

**Authors:** Thomas Desmet, Lauren Van Haesendonck, Sophie Vermeire, Eline van Overbeeke, Steven Simoens, Isabelle Huys

**Affiliations:** ^1^ Department of Pharmaceutical and Pharmacological Sciences, Clinical Pharmacology and Pharmacotherapy KU Leuven, Leuven, Belgium; ^2^ Healthcare Management Centre, Vlerick Business School, Ghent, Belgium

**Keywords:** duchenne muscular dystrophy, attribute development, semi-structured interviews, gene therapy, patient preferences, caregiver perspectives, qualitative research

## Abstract

**Background:**

Duchenne muscular dystrophy (DMD) is an X-linked degenerative muscle disease with no curative treatment available to date. The current long-term use of corticosteroids is associated with severe adverse effects. With the progress of promising gene therapy for DMD, this research aims to identify the key characteristics that matter most to patients, develop attributes for a subsequent quantitative preference study, ultimately aimed to inform future market access and clinical decision-making on gene therapy.

**Methods:**

A literature review was conducted, followed by semi-structured interviews with DMD patients and caregivers to explore their preferences regarding DMD treatment benefits and side effects and gene therapy as a promising treatment option. A ranking exercise helped reveal the most important treatment characteristics, forming the basis for the first step of a structured, four-step attribute and level development process: (1) attribute identification, (2) attribute selection, (3) attribute description, and (4) level development. The forthcoming six attributes and levels were determined by applying six inclusion and exclusion criteria aligned with PREFER guidelines and reaching consensus within an international multidisciplinary advisory board comprising patient representatives, clinicians, and preference method experts.

**Results:**

A total of thirteen interviews were conducted with seven DMD patients and eleven caregivers. The literature review and interviews resulted in the identification of 48 unique disease and treatment characteristics. Furthermore, they revealed a high willingness of caregivers of especially younger children to consider gene therapy in a clinical trial setting, and that the primary treatment characteristics valued by patients and caregivers are related to muscle and heart function, and the impact on self-care activities, independence. The final attributes are patient-friendly, clinically relevant and meaningful to patients, with descriptions that are as brief as possible: the type of therapy, effect on life expectancy, risk of life-threatening side effects related to the therapy, years that ventilatory support can be postponed, number of years maintaining current physical functioning, and years and number of patients in which that therapy has been studied.

**Conclusion:**

This study identified the treatment characteristics most important to DMD patients and their caregivers and translated them into six key attributes with corresponding levels. It underscores the practical value of qualitative research and patient engagement in ensuring that attributes and level development for future quantitative preference elicitation studies remain clinically relevant and aligned with patient priorities.

## 1 Introduction

**FIGURE 1:**
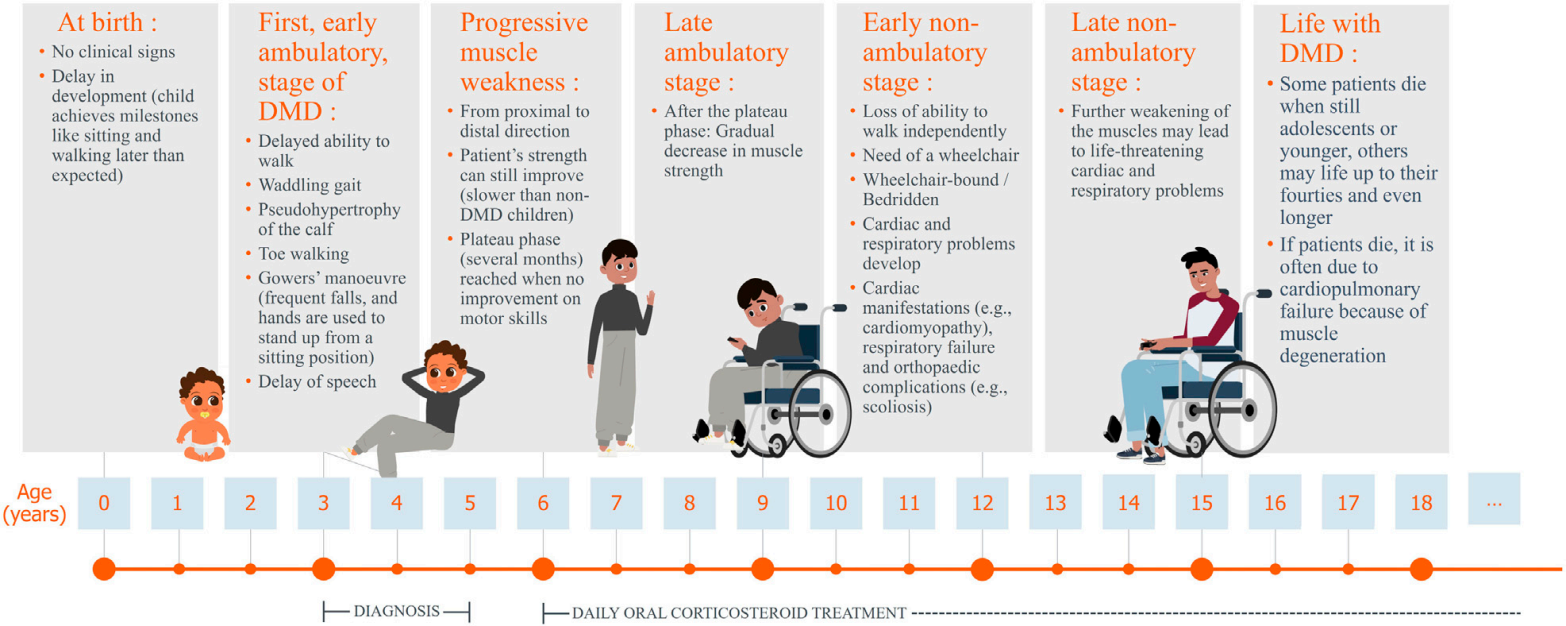
Lifecycle of a patient with Duchenne muscular dystrophy, including typical symptoms.

Duchenne muscular dystrophy (DMD) is a rare disease, with a worldwide incidence rate of one in 3500 to one in 5000 live male births (Shieh, 2018; Sun et al., 2020; Verhaart and Aartsma-Rus, 2019; Chamberlain and Chamberlain, 2017; Mah, 2016). The prevalence of DMD is approximately 7.1 per 100,000 male individuals (Crisafulli et al., 2020). DMD primarily affects boys because it is an X-linked neuromuscular disorder, females are mostly asymptomatic carriers (Yiu and Kornberg, 2015). The disorder is characterised by progressive muscle weakness (Chang et al., 2016; Crone and Mah, 2018; Reinig et al., 2017) due to mutations in one of the largest human genes, the DMD gene, which encodes the dystrophin protein (Falzarano et al., 2015; Suthar and Sankhyan, 2018). This results in a lack of functional dystrophin in skeletal, heart, lung, and other muscle cells, as well as neurons, leading to muscle fibre degeneration, contributing to the symptoms of DMD patients. Minor symptoms appear early in life, with noticeable difficulties in walking and climbing stairs emerging around the age of three to four. As the disease progresses, young boys typically lose their ability to walk and require a wheelchair, often accompanied by severe skeletal deformities that persist lifelong. In adolescence, impaired arm (upper limb) function becomes evident and significantly affects daily activities. Between the ages of 15 and 20 years, many patients require nighttime ventilation, which gradually progresses to 24-h respiratory support in their twenties. The disease reaches a critical phase between the late 20s and mid-30s, when mortality rates are high, primarily due to respiratory or cardiac complications (Walter and Reilich, 2017) (Figure 1).

To reduce and control these symptoms, the current standard of care involves a multidisciplinary approach to slow disease progression and facilitate rehabilitation (Shieh, 2018; Crone and Mah, 2018; Messina and Vita, 2018; Ke et al., 2019). Cardiac and respiratory management is essential to increase life expectancy (Shieh, 2018; Crone and Mah, 2018; Falzarano et al., 2015; Suthar and Sankhyan, 2018; Arora, 2019; Davis et al., 2015; Guglieri et al., 2017; Birnkrant et al., 2018a; Kostek and Gordon, 2018) and improve quality of life (QoL) (Shieh, 2018; Yiu and Kornberg, 2015; Reinig et al., 2017). The multidisciplinary approach consists of pharmacological management with corticosteroids, alongside supportive care aimed at reducing fibrosis and inflammation, improving muscle function and protection, restoring cellular energy, enhancing heart function, and regulating the calcium balance. Physiotherapy is also an essential component to preserve mobility and independence, as is psychological counselling to improve the mental wellbeing of both patients and their families to cope with the emotional challenges of the disease. These current therapies have shown key benefits, including improved muscle strength and function, prolonged ambulation, delayed onset of cardiomyopathy, improved pulmonary function, and slowed progression of scoliosis (Ke et al., 2019; Guglieri et al., 2017; Birnkrant et al., 2018b; Matthews et al., 2016). The current multidisciplinary management of the disease has extended the life span of patients with DMD from their early twenties to their forties (Reinig et al., 2017; Guglieri et al., 2017). However, DMD remains incurable, and the side effects associated with the chronic use of current therapies cannot be overlooked. These include reduced growth, weight gain, behavioural changes, hirsutism, osteoporosis, hypertension, immune suppression leading to life-threatening infections, and cataracts (Reinig et al., 2017; Guglieri et al., 2017; Birnkrant et al., 2018b; Matthews et al., 2016; Beytía Mde et al., 2012; McDonald et al., 2012; Manzur et al., 2004).

Innovative therapies under investigation, such as exon skipping, stop codon readthrough, gene therapy, and CRISPR-Cas9 can potentially fill in some of the unmet medical needs (Sun et al., 2020; Verhaart and Aartsma-Rus, 2019; Reinig et al., 2017; Suthar and Sankhyan, 2018; Messina and Vita, 2018; Arora, 2019; Verhaart and Aartsma-Rus, 2012; Iannaccone and Nanjiani, 2001). Gene therapies targeting the root cause of the disease hold this promise by introducing a functional micro- or mini-dystrophin gene (transgene) (Annexstad et al., 2014; Hoffman, 2020). This transgene is introduced into muscle cells using adeno-associated viral (AAV) vectors through a one-time systemic administration. The goal is to enable patients to express a partially functional dystrophin, and thus achieve slower disease progression over time (Barthélémy and Wein, 2018; Shimizu-Motohashi et al., 2016; Liu et al., 2018; Aguti et al., 2018; Thorne et al., 2018). In June 2024, the FDA approved the first gene therapy for ambulatory DMD patients aged ≥4 years: delandistrogene moxeparvovec (Elevydis^®^). For non-ambulatory patients, treatment has been approved under accelerated approval, although further clinical trial (CT) data are still required. In Europe, no gene therapy for DMD has been approved yet.

While gene therapy presents a promising new approach, it remains associated with uncertainties regarding its long-term benefits, risks, and acceptability among patients and caregivers. Given that treatment decisions in DMD are preference-sensitive, understanding which treatment characteristics matter most to patients and caregivers is crucial for optimizing future therapeutic development, regulatory decision-making, and healthcare policies (Gärtner et al., 2019; van Overbeeke et al., 2019; van Overbeeke et al., 2021a; Hollin et al., 2017). This research aims to identify and define the most relevant treatment characteristics and translate them into attributes and levels for use in future quantitative patient preference studies. The insights gained will help ensure that treatment evaluations reflect the priorities of patients and caregivers, ultimately guiding more patient-centered decision-making in DMD treatment development.

## 2 Methods

### 2.1 Defining disease and treatment characteristics

The first part of this study consists of an interview study to define disease and treatment characteristics. This part was based on the protocol of the qualitative phase of the Patient preferences to Assess Value IN Gene therapies (PAVING) study in haemophilia (van Overbeeke et al., 2021b; van O et al., 2021). Ethical approval for the semi-structured interviews was granted by the ethics committee research UZ/KU Leuven (S64990) on 4th of February 2021. The interview guide included open questions (Supplementary Appendix 1) and was developed based on a literature review of clinical trials and patient preference studies (Supplementary Appendix 2). Participants could indicate their willingness to be treated or to let their child be treated with gene therapy, could share their perspectives towards long-term efficacy, safety, and uncertainties. The interview guide was reviewed by several healthcare professionals (HCPs) and parents of boys with DMD. Pilot interviews in both Dutch and French were performed, both pilot interviews were included in the final analysis as no major changes were deemed necessary.

#### 2.1.1 Participant recruitment

French or Dutch-speaking Belgian DMD patients aged 16 years and older and adult caregivers were invited by SV via the Duchenne Parent Project (DPP) Belgium, or via the neuromuscular reference centres (NMRCs) of UZ Leuven and UCL Saint-Luc. Due to the paediatric nature, treatment decisions for underage children are often made by caregivers. A caregiver is defined as “a spouse, partner, legal guardian, close relative, or other adult close to the family, living either in the same house or in contact with the DMD patient at least four times per week for at least 1 hour or more per day” (Jimenez-Moreno et al., 2020). Respectively, assent and consent were retrieved before letting eligible participants fill out an online demographics and characteristics questionnaire (Supplementary Appendix 3) or take part in the interviews. Both ambulatory and non-ambulatory patients and their caregivers could participate. The health literacy of participants was assessed using three health literacy screening questions developed by Chew et al. Based on a 5-point Likert scale, an average score of ≤2 indicates inadequate health literacy, while a score of >2 suggests adequate health literacy (Chew et al., 2004; Fransen et al., 2011).

#### 2.1.2 Conduct of the interviews

Between February 2021 and May 2021, individual interviews with adult patients and caregivers, as well as dyadic interviews, in which an underage person with DMD was accompanied by an adult caregiver, were conducted. To prioritise the patient’s own opinion in these dyadic interviews, caregivers were asked to respond only after the patient had given their answer. The semi-structured interviews were conducted in the participant’s native language via phone, Skype or Zoom, and lasted approximately 1 hour. To ensure a common knowledge level at the beginning of the interviews, participants received visually supported information on the disease, available treatment options, and the goal and mechanism of action of gene therapy.

As proposed by Kerr et al., a saturation table (Supplementary Appendix 4) was created to determine when no new themes emerged in subsequent interviews, indicating that saturation had been reached (Kerr et al., 2010). Next to the open questions, the interviewees were asked to rank seventeen categorised treatment and disease characteristics (Supplementary Appendix 5) identified in the literature (top-down) and characteristics that they mentioned spontaneously (bottom-up) (van Overbeeke et al., 2021b; Soekhai et al., 2019; George and Apter, 2004; Coast et al., 2012; Peters and Halcomb, 2015). Additionally, they were asked to rank six characteristics from the least important to the most important when considering gene therapy. To conclude, two cases based on preliminary gene therapy trial data were presented to explore what patients and caregivers value when considering gene therapy or corticosteroids for DMD (Verhaart and Aartsma-Rus, 2012; Okada and Takeda, 2013). The cases covered an eight-year-old boy diagnosed with DMD, who did not need a wheelchair yet (Supplementary Appendix 6) and included details on the route of administration, treatment regimen, potential effects and expected side effects.

#### 2.1.3 Data analysis

Descriptive statistics were used to analyse the demographics and results from the ranking exercise. Using framework analysis, the qualitative interview data were analysed in Nvivo Software (Gale et al., 2013). This was made possible by having the interviews recorded and transcribed *at verbatim* before being permanently removed. Data were pseudonymised to ensure confidentiality. Codes were created in a deductive way, based on the interview guide, and inductive way, arising throughout the process of analysing the transcripts, and subsequently organised in a coding tree (Supplementary Appendix 7).

Based on the final coding tree, a framework matrix was constructed to compare the opinions of different participants, separately for patients and caregivers. To ensure comprehensive reporting, the Consolidated Criteria for Reporting Qualitative Research (COREQ) checklist, a 32-item checklist for interviews, was used (Supplementary Appendix 8) (Tong et al., 2007).

### 2.2 Attribute and level development

**FIGURE 2:**
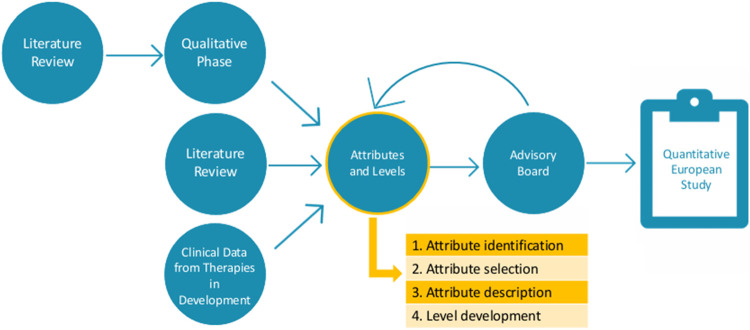
Visualization of the study design.

The second part of this study consists of attribute development and level determination. Ethical approval by the ethics committee research UZ/KU Leuven (S66104) was obtained on the 19th of January 2022. Following the PREFER recommendations (Consortium, 2022), the results of the first part were amended by an additional literature review and CT data to ensure all important characteristics were identified before further attribute development (Figure 2). To guide this second part, an international multi-stakeholder advisory board was established, consisting of patient representatives, clinicians, and preference method experts.

The attribute development process was divided into four steps: (1) characteristic identification, (2) characteristic selection, (3) attribute description, and (4) level development.

#### 2.2.1 Characteristic identification

The first-hand insights from the interviews played a crucial role in identifying and incorporating context-specific characteristics, not always described in the literature, while also capturing the perspectives and experiences of the target population, i.e., through the ranking exercise and via spontaneously mentioned characteristics (Coast et al., 2012; Abiiro et al., 2014; Rydén et al., 2017).

#### 2.2.2 Characteristic selection

The appropriate selection of characteristics is essential to minimize bias and error (Rydén et al., 2017), therefore, the attribute selection process was guided by six criteria: attributes (i) must relate to DMD treatment, (ii) must be patient-centered and meaningful for patients, even if the characteristics were derived from caregivers, (iii) must be derived from CTs, selected literature, qualitative studies, or expert opinions from medical professionals or decision-makers, (iv) must be selected as informed by patients, patient representatives, and experts, (v) must have minimal to no overlap with other attributes, and (vi) must be expressed numerically to be used in a probabilistic threshold technique survey (Janssens et al., 2019; Hauber and Coulter, 2020).

These selection criteria allow for a systematic reduction of the identified characteristics while ensuring that the final attributes remain patient-relevant and methodologically appropriate. In addition to the selection criteria, the frequency with which characteristics are reported in the literature and their perceived importance according to the interviews, and ranking exercise are considered. A limit to select of up to six attributes was set to ensure that the subsequent questionnaire remains cognitively feasible for the target population (van Overbeeke et al., 2021).

#### 2.2.3 Attribute description

Next, descriptions of the selected attributes were made using patient-relevant terminology in close dialogue with the advisory board in multiple meetings. Linking back to the transcripts of the semi-structured interviews ensured that the wording closely reflected the language used by patients and caregivers, which often differs from terminology found in the literature. As a result, the attributes are described in a patient-friendly, clear, and concise way.

#### 2.2.4 Level development

In the last step, the attributes were accompanied by clinically plausible and patient-relevant levels, selected on current clinical evidence (clinicaltrials.gov) and expert consultation. For each attribute, up to seven scientifically grounded levels are assigned, which allow us to influence how appealing the treatment profiles are. While the treatment profiles remain hypothetical, basing them on existing CT data, literature, and clinical experts allowed us to anticipate future decisions that patients may need to make (van Overbeeke et al., 2021; Hauber and Coulter, 2020).

## 3 Results

### 3.1 Study population

A total of thirteen interviews (six in Dutch and seven in French) were conducted. Typically, interviews are conducted until data saturation is reached, meaning that no new themes emerge from additional interviews. While in our study, data saturation was reached early, after only three interviews (Supplementary Appendix 4), additional interviews were conducted to strengthen the diversity and validity of the data. A total of eleven caregivers and seven patients participated in interviews, their self-reported characteristics are summarized in Table 1. Five dyadic interviews were held, where patients were accompanied by their caregivers. The two oldest patients, aged 29 and 39, as well as six caregivers participated individually. The group of caregivers provided information about twelve patients as one caregiver had two sons with DMD. All interviewed caregivers were mothers, except for one who was the sister of a person with DMD. Half of the mothers were identified as carrier of the disease.

**TABLE 1:** Self-reported characteristics of caregivers and patients.

Characteristics	Caregivers (N = 11), reporting on 12 patients	Patients (N = 7)
Age caregiver (years) Mean (SD)	41.3 (10.0)	-
Relation to the patient n (%)		-
Mother	10 (90.9)	-
Close relative	1 (9.1)	-
Mother carrier n (%)		
Yes	5 (50.0)	-
No	5 (50.0)	-
Gender n (%)		
Female	11 (100)	0 (0)
Male	0 (0)	7 (100)
Residence respondent n (%)		
Flanders	6 (54.5)	3 (42.9)
Wallonia	4 (36.4)	3 (42.9)
Brussels	1 (9.1)	1 (14.3)
Age patient (years)		
Mean age (SD)	12.1 (6.3)	23.3 (7.9)
Min - max	4.5–21	18–39
Age patient at diagnosis (years)		
Mean age (SD)	2.1 (2.0)	2.5 (2.2)
Min - max	0.25–6	0–6
Mobility of the patient n (%)		
Patient can walk very well	4 (33.3)	1 (14.3)
Long distance and stairs are difficult	6 (50)	2 (28.6)
Patient can take some steps (with or without support)	0 (0)	1 (14.3)
Patient cannot walk	2 (16.7)	3 (42.9)
Corticosteroid treatment n (%)		
Never	2 (16.7)	2 (28.6)
Both in the past and currently	10 (83.3)	5 (71.4)
Patients with experience in clinical trial recruitment n (%)		
Yes	8 (66.7)	6 (85.7)
No	4 (33.3)	1 (14.3)
Satisfaction of current treatment n (%)		
Very satisfied	1 (9.1)	0 (0)
Satisfied	3 (27.3)	3 (42.9)
Neutral	6 (54.5)	4 (57.1)
Not satisfied	1 (9.1)	0 (0)
Health literacy score n (%)		
Adequate health literacy	10 (90.9)	6 (85.7)
Inadequate health literacy	1 (9.1)	1 (14.3)

The mean age at which DMD was diagnosed in patients was 2.3 years old (minimum 5 days after birth, maximum 6 years old). All DMD patients, apart from the two youngest, were treated with corticosteroids as part of the multidisciplinary treatment. They had been receiving corticosteroids in the past and were still receiving corticosteroids at the time of the interviews. While both caregivers and patients reported being neutral to satisfied with current corticosteroid treatment in the pre-interview questionnaire, the interviews revealed more nuanced and often critical lived experiences. Fourteen out of nineteen patients were familiar with participating in a CT. Regarding health literacy, one caregiver and one patient had an inadequate score (≤2).

#### 3.1.1 Baseline knowledge of gene therapy

**FIGURE 3:**
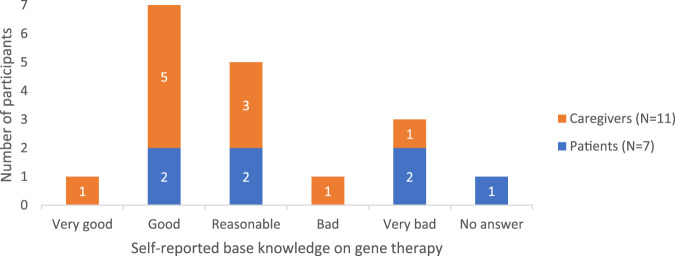
Heterogeneous self-perceived knowledge rating on gene therapy.

Among participants, there was variation in the knowledge about treatment options like gene therapy, from very superficial knowledge to a good understanding. This was also reflected in their self-assessed knowledge level ratings (Figure 3).

Although interviewed caregivers were aware of the existence of gene therapy, “*heard about gene therapy for a while*”, and some had a fair idea about it, others confused it with other innovative therapies such as exon skipping and CRISPR/Cas9. Only a few could share some knowledge about the preclinical results, i.e., in dog models, and ongoing CTs. Three caregivers demonstrated some awareness of technical aspects, though they made some critical medical inaccuracies such as stating: “*DNA of micro-dystrophin is built in a virus [viral vector]*” and that with this, gene therapy “*uses viruses [viral vectors] to transport”* the gene to the muscle, which would result in dystrophin production. Two caregivers specified that adenoviruses [adeno-associated viral (AAV) vectors] are used in case of DMD. Moreover, one caregiver was aware that antibodies against the virus could already be present “*because those AAVs are also effectively already just naturally present as viruses*” and additionally also mentioned that for now patients can only be treated once with gene therapy because “*antibodies are produced, which stays in your immune memory*”. Finally, two caregivers noted that gene therapy differs from CRISPR and other gene-editing techniques, emphasizing that there is “*no real tampering with the genes*”. As a result, they perceived gene therapy to be a safer option. Overall, patients had a more superficial idea about gene therapy: “*It has something to do with genetic material”* and that “*the goal is to repair genes*”. One patient explained that gene therapy involves injecting a gene, while another elaborated that its purpose is “*to play a replacement role for the DNA that we, as Duchenne patients, lack*”.

#### 3.1.2 Sources of information for patients and caregivers

Patients mainly looked online to get answers to their questions, next came the patient organisation Duchenne Parent Project (DPP) Belgium, their parents or their treating physician or geneticist. Most caregivers also relied on online sources for information but also recognised the value of consulting with other Duchenne families, attending scientific congresses, reading scientific publications, or getting advice from their Neuromuscular Reference Center (NMRC). Two caregivers received information directly from their physicians, while others gained insights through (international) conferences, scientific literature, or patient associations such as DPP Belgium or Téléthon. One caregiver additionally expressed openness to sharing treatment experiences, seeking information, or asking questions in small discussion groups facilitated by a physician.

### 3.2 Gene therapy information and education

Although, the education aspect prior to the interviews was limited to what was needed to participate in the interviews, it was generally well-received. Both patients and caregivers were eager to learn and know more about gene therapy and tools, e.g., screening for pre-existing antibodies, subtypes of AAVs used in CTs, as well as the results of gene therapies in CTs. Participants indicated that the general information about the benefits and side effects, as well as the practical information about administration and follow-up on gene therapies was comprehensive and easy to understand. The visualisations of complex processes such as the mechanism of action, the concept of antibodies against the vector, and the possibility of pre-existing antibodies, helped to clarify misconceptions among participants. However, some information had to be repeated before participants confirmed their understanding. Participant feedback on the educational material suggests further refinement is needed. For example, it needs to emphasize that gene therapy “*does not change the genetic make-up of patients”* and that it is likely that the disease symptoms “*will change from Duchenne to Becker disease”* as dystrophin production will be partially improved, but that damage caused by DMD is irreversible, even with gene therapy.

#### 3.2.1 Perspectives on gene therapy: motivations, concerns, and uncertainties

Patients and caregivers were positive towards its potential benefits such as physical effects and quality of life on which they focused most. Although there were concerns about the efficacy-related and safety-related uncertainties, one patient-caregiver pair stood out as the only pair not in favour of pursuing gene therapy. Among the remaining participants, three patients and six caregivers were willing, while three patients and four caregivers were very willing to use gene therapy. A 21-year-old ambulant patient and six caregivers spontaneously mentioned being willing to use gene therapy in a CT setting, whereas six patients and five caregivers would not. It was clear that there is a real need for a new treatment. One of the caregivers also worded this explicitly as:

“The need is high, really high.”

This exemplified the hope that patients and caregivers share for gene therapy in offering muscle strength stabilisation to offer the patient an independent life of good quality, wherein the patient can keep doing what they can today, e.g., no need for a wheelchair. Others linked this to a good quality of life and independence. Although patients would appreciate stabilisation, they also hope for muscle strength improvement. Besides, caregivers also hope gene therapy can be effective on heart and lung function (e.g., less shortness of breath). Moreover, patients would like to stop the intake of corticosteroids to no longer experience the side effects of reduced growth.

Caregivers reported that the patients’ disease status strongly influences their treatment decisions, particularly their willingness to take risks. When a patient is doing well, caregivers feel reassured about their choice, while new side effects could increase doubts and negatively impact their choices. They also weigh the benefit-risk balance, considering factors like treatment duration and time commitment, for example, brief daily administration versus a few hours once a month. Finally, many also emphasized trust in their physicians when making decisions:

“*The doctors give you comfort and the feeling that you are in good hands*.”

Whereas patients did not mention any reasons to refrain from gene therapy, caregivers mentioned concerns around side effects, the high dose of corticosteroids required before gene therapy, the immune response limiting the treatment to a one-time option, exclusion from future CTs, and hesitancy to take risks when the child is in stable condition. An overview of participants’ individual top three characteristics influencing treatment choice can be found in Supplementary Appendix 9.

Eight of the eleven caregivers said that they would not refrain from gene therapy because of uncertainties regarding its efficacy. Moreover, two caregivers stated not to overthink the uncertainties, but rather live in the moment as we cannot predict what the future brings. Two patients admitted feeling somewhat frightened by uncertainties, noting that study findings are often presented as breakthroughs. They emphasized the need for more cautious communication to avoid spreading false hope among patients and their families. Four other patients indicated that, despite efficacy-related uncertainties, they would still opt for gene therapy.

Patients’ opinions regarding long-term safety and efficacy uncertainties were similar. In contrast, caregivers were more concerned about uncertainties about long-term safety than efficacy. However, one caregiver specified that a disease stabilisation or improvement would outweigh the uncertainty of long-term safety concerns. Two patients had no opinion regarding the safety-related uncertainties of gene therapy. One patient admitted feeling somewhat scared but chose not to focus on it. Lastly, one patient and one unrelated caregiver compared the gene therapy uncertainties with those surrounding the development of COVID-19 vaccines. One caregiver who would let her child be treated with gene therapy in a CT also hoped the patient would not regret the parents’ decision once they are old enough to make their own choices.

Quite contradicting, half of the caregivers who expressed willingness towards gene therapy in CT setting, require proven efficacy for a minimum of 6 months, up to one, two, five, 10 years or even lifelong efficacy, only three caregivers required no prior data. Two patients required one to 5 years of proven efficacy, while two others required at least 10 years of data. Serious side effects, the temporary high intake of corticosteroids, or not being able to participate in other trials were factors that partially tempered the willingness to use gene therapy. Also, regarding safety data, the caregivers willing to enrol their child in CTs did not require a minimum number of proven years, as long as it did not pose a life-threatening risk. Others, however, required ten to 20 years of safety data. One caregiver, confident in the benefits of gene therapy, considered 6 months of safety data sufficient. Another caregiver struggled to articulate a specific timeframe but reasoned:

“If there is no serious side effect in the first few months, I do not think there will be any in x-number of years.”

For caregivers, safety-related uncertainty did not outweigh the willingness to use gene therapy as long as the therapy would be effective.

Patients indicated they did not need any or only one or 2 years of proven safety, “*it just has to be safe”.* There was one patient, together with his mother, who explicitly expressed the need for lifelong safety data.

#### 3.2.2 Feeling towards the presence of antibodies

Regarding pre-existing antibodies, one caregiver and one patient had no opinion. Several caregivers and patients expressed feelings of frustration with the idea that patients with pre-existing antibodies cannot be treated with gene therapy. Meanwhile, others accepted this or expressed hope for other vectors in the future that would not be affected by pre-existing antibodies.

### 3.3 Characteristic ranking exercise

Participants were asked to individually rank the six characteristics they considered most important in deciding whether to accept or refrain from gene therapy. These were selected from a combined list of seventeen predefined and spontaneously mentioned characteristics. The effect on muscle function was the most frequently identified characteristic in the literature (n = 5) and was also ranked as the most important by both DMD patients and caregivers. The second most frequently identified characteristics were the effect on heart function, the effect on lung function, uncertainty about treatment benefits, and risk of death (n = 3). The list also reflected the concerns about safety-related uncertainties and the focus on previously experienced side effects of current therapy. Table 2 summarizes the ranking of top five characteristics as well as the ones that were deemed less important in the decision-making process for gene therapy. One of the caregivers ranked “the patient’s health” as the most important, explaining this included the effect on the heart, lung, and muscle function. For a patient ‘performing self-care activities’ influenced the ranking of ‘the impact on the patient’s social life’. One patient and three caregivers considered all factors important. Overall, the mechanism of action and route of administration were ranked as the least important factors. A detailed overview of the individual results is provided in Supplementary Appendix 5.

**TABLE 2:** Overview of the five most highly rated characteristics and ten less prioritized ones.

Ranking	Caregivers (n = 8)	Patients (n = 6)
1	Effect on muscle function	Effect on muscle function
2	Effect on the heart	Effect on the heart
3	Impact on self-care activities, independence	Impact on self-care activities, independence
4	Effect on lung function	Impact on patients’ social life
5	Impact on patients’ social life	Risk of short-term side effects; Effect on cough strength
6	Period of follow-up (n = 5)	Route of administration (n = 3)
7	Frequency of follow-up (n = 4)	Mechanism of action (n = 2)
8	Route of administration (n = 3)	Period of follow-up (n = 1)
9	Mechanism of action (n = 2)	Frequency of follow-up (n = 1)
10	Impact on the caregivers’ social life (n = 2)	Impact on the caregivers’ social life (n = 1)
11	Probability corticosteroids can be stopped (n = 1)*	Probability corticosteroids can be stopped (n = 1)*
12	Dose frequency (n = 1)	Dose frequency (n = 1)
13	Impact on the patients’ social life (n = 1)	Impact on the patients’ social life (n = 1)
14	Risk of short-term side effects (n = 1)	Risk of short-term side effects (n = 1)
15		Uncertainty of long-term side effects (n = 1)

*has never used corticosteroids.

### 3.4 Cases: symptomatic treatment versus gene therapy

In the first case, daily oral corticosteroids were compared to a single intravenous gene therapy. Corticosteroids slowed muscle deterioration but came with long-term side effects, while gene therapy offered the possibility of stabilizing or improving muscle strength, though its long-term efficacy and side effects were uncertain. One patient-caregiver pair chose corticosteroids, believing oral administration was *“easier for an eight-year-old child”* and that gene therapy’s side effects were too severe. Caregivers found the decision challenging due to uncertainties with the presented gene therapy; however, they chose it, hoping it would preserve the muscle function and mobility of the patient. Other participants convincingly opted for gene therapy, citing its potential to stabilize or improve muscle strength. Three caregivers and two patients noted that corticosteroids only treated symptoms without preventing further decline, whereas gene therapy could provide more lasting benefits. Two caregivers and one of the patients reasoned that gene therapy can buy you time and, if necessary, it is still possible to switch back to corticosteroid treatment.

In the second case, corticosteroids were again compared to gene therapy, which this time led to temporary stabilization before muscle strength declined. As in the first case, the same patient and accompanying caregiver preferred corticosteroids due to concerns about gene therapy’s side effects, and route of administration. Except for them, no one thought that intravenous administration would be a problem for children; even more, the single administration was seen as an advantage. The remaining eight caregivers and five patients favoured gene therapy, seeing even temporary stabilization as valuable and believing that if needed, they could switch back to corticosteroids. Patients were mostly focused on muscle strength improvement when discussing the cases.

Overall, most participants chose gene therapy in both cases, believing its potential benefits outweighed the side effects of long-term corticosteroid use. Interestingly, patients focused on the short-term side effects, and three adult patients scored cough strength as important, whereas caregivers considered long-term cardiopulmonary complications, the leading cause of death in DMD, as their primary concern. All caregivers, except for one, neglected cough strength as their young children had not yet experienced respiratory issues.

### 3.5 Development of attributes and levels

#### 3.5.1 Characteristic identification

**FIGURE 4:**
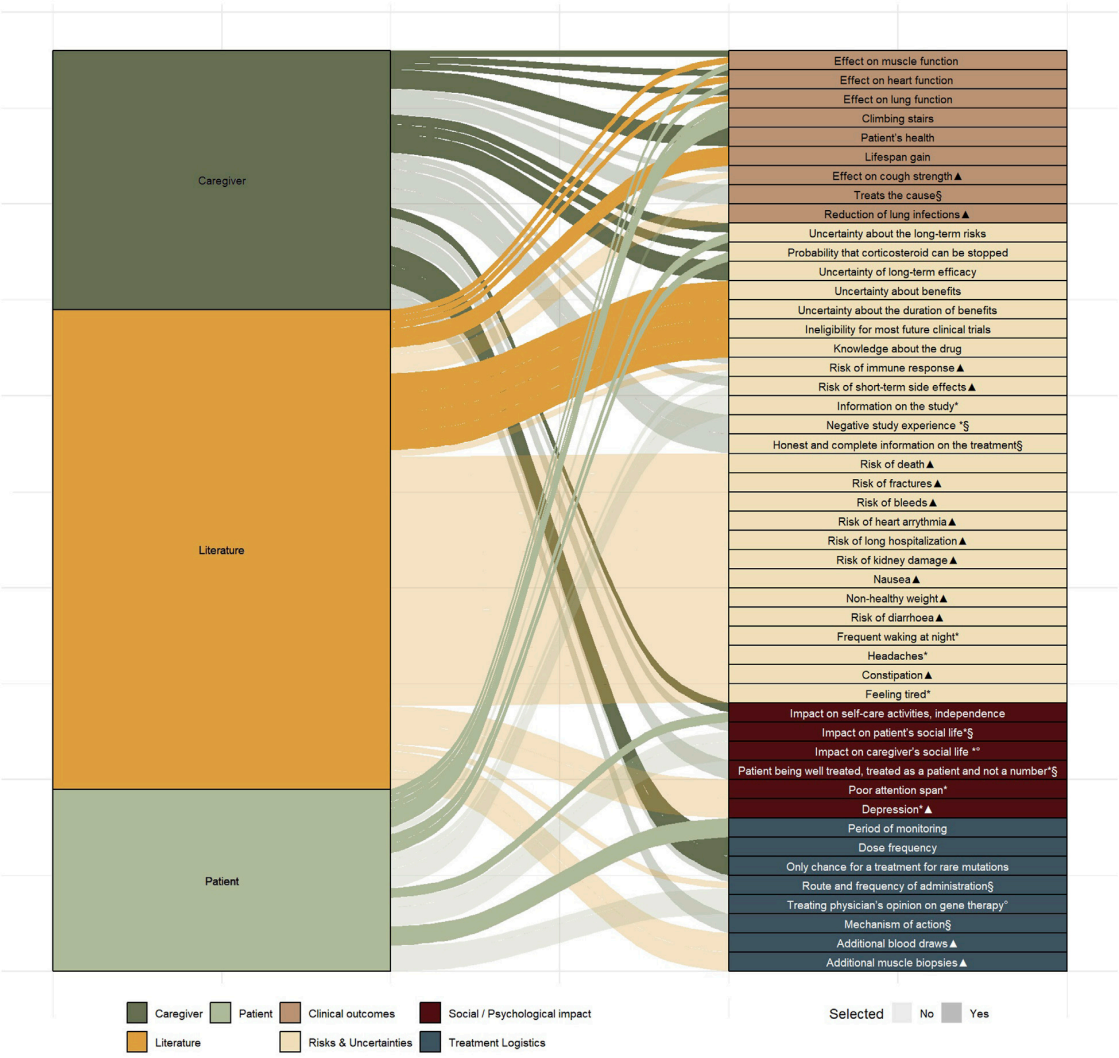
Uniquely identified characteristics (n = 48) from literature (n = 30) and from the semi-structured interviews (n = 26) mapped across four domains Markings indicate characteristics that did not meet the predefined selection requirements: *Not treatment-related* (*), *Not quantifiable* (§), *Not patient-centred* (°), *Overlapping characteristic* (▲).

To develop attributes, the 30 characteristics identified from the literature and 26 from the semi-structured interviews were compiled, and duplicates were removed in consultation with the advisory board. Of the total 56 identified characteristics, 48 unique characteristics emerged (Figure 4 and Supplementary Appendix 10).

#### 3.5.2 Characteristic selection

Based on the six selection criteria, described in the methods section, a preselection of characteristics was made. Thirty-one characteristics did not fulfil all selection criteria, whereas seventeen did. Of these seventeen, six characteristics were selected by the advisory board for further attribute development, which took into account the ranking exercise (Table 2) performed in the interview phase. Certain characteristics, such as, e.g., climbing stairs and impact on self-care activities and independence were grouped under a single attribute “effect on mobility” due to their close interrelation. While mobility and muscle function influence each other, they may hold different values for patients and caregivers, therefore, both were retained as separate attributes.

#### 3.5.3 Attribute description

**FIGURE 5:**
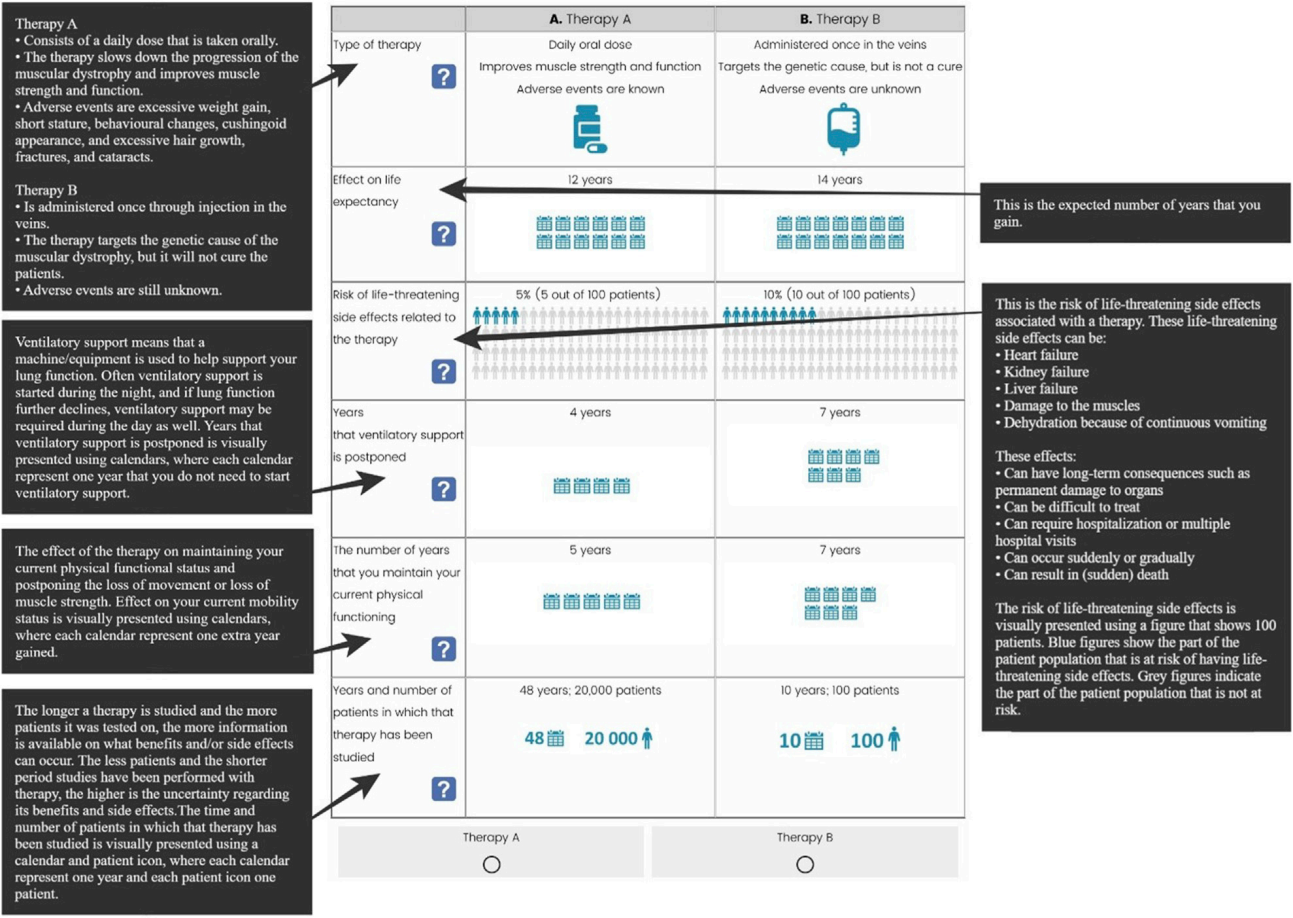
Attributes and their respective attribute description.

Patient representatives provided valuable input, particularly in refining the lung function attribute. Since not all patients have experienced lung problems, and baseline lung function can significantly differ across disease stages, phrasing the attribute as “a delay in the onset of lung function decline” or as “the percentage of lung function patients hoped to attain” was considered, respectively, irrelevant or too complex. The final developed attributes are patient-friendly and easily understandable, with descriptions that are as brief as possible: the type of therapy, its effect on life expectancy, risk of life-threatening side effects related to the therapy, years that ventilatory support is postponed, the number of years that you to maintain your current physical functioning, and years and number of patients in which that therapy has been studied (Figure 5).

#### 3.5.4 Level development

The advisory board agreed on the final attributes as well as the levels, which were based on the at the time most up-to-date clinical evidence that was publicly available (Table 3).

**TABLE 3:** Threshold series showing participant switching points between fixed treatment reference (therapy A) and therapy B across increasing or decreasing attribute levels.

	Therapy A	Therapy B								
Levels		Most appealing				Baseline	Least appealing			
		9	A	B	C	D	E	F	G	8
Type of therapy	- Daily oral dose - Improves muscle strength and function - Adverse events are known									
						- Administered once in the veins - Targets the genetic cause, but is not a cure - Adverse events are unknown				
Effect on life expectancy	12 years	>16 or no therapy		16	15	14	13	12		0–11
Risk of life-threatening side effects related to the therapy	5% (5 out of 100)	<1%		1%	5%	10%	15%	13%		14%–100%
Years that ventilatory support is postponed	4 years	>10	10	9	8	7	6	5	4	1–3
The number of years that you maintain your current physical functioning (ambulant)	3 years	>5 or no therapy		5	4.5	4	3.5	3		0–2.5
The number of years that you maintain your current physical functioning (non-ambulant)	5 years	>9 or no therapy		9	8	7	6	5		0–5
Years and number of patients in which that therapy has been studied	48 years 20,000 patients	>15 >300	15 300	14 250	11 150	10 100	8 15	7 10	3 3	0–3 0–3

Levels represent increasing or decreasing desirability of a treatment attribute, with D as the baseline. Participants may shift preference away from baseline level D toward more (B) or less (F) favourable levels. Moving left (D→B→A/C) reflect more appealing outcomes than baseline; moving right (D→F→E/G) reflects less appealing outcomes. Dropdown thresholds (Crone and Mah, 2018; Chang et al., 2016) indicate when a participant’s preference did not switch even at the most favourable level (9 > B or A) or at the least favourable level (8 < F or G), respectively.

## 4 Discussion

With gene therapy trials in DMD progressing and even getting marketing authorisation in the US, hope for European DMD patients to access treatment that goes beyond symptomatic treatment is growing. Therefore, with the patients’ perspective progressively becoming more important in medical and regulatory decision making, patient preference studies are rapidly increasing (Hauber and Coulter, 2020; Hollin et al., 2020). Since DMD primarily affects children, decisions about treatments are often made by parents or caregivers. However, it is known that preference heterogeneity exists among patients in different stages, and unique individual experiences. Unlike previous preference research in the US and UK, this study did also take into account the opinion of non-adult patients using dyadic interviews and took into account the most recent gene therapy insights as not only several gene therapies for various diseases have reached the market, but also CTs for gene therapy in DMD has made significant progress (Peay et al., 2014; Landrum et al., 2019; Hollin et al., 2016; Heslop et al., 2021). Additionally, few papers demonstrate full transparency in the attribute and level development process, or this description appears inadequate (Coast et al., 2012; Abiiro et al., 2014; Rydén et al., 2017; Hollin et al., 2020). Therefore, this study, constituting the crucial first steps in qualitatively exploring those perspectives to prepare a future survey to elicit them quantitatively, answers the critique of lack of transparency regarding attribute and level development with a rigorous development process (van Overbeeke et al., 2019; Soekhai et al., 2019; Hollin et al., 2020; Settumba et al., 2019).

### 4.1 Baseline knowledge and information retrieval

The diverse baseline knowledge levels about DMD gene therapies among participants highlighted the ongoing need for accessible, tailored information. In general, both caregivers and patients showed great interest in additional information on gene therapy, which aligns with findings from previous studies (Heslop et al., 2021; Aiyegbusi et al., 2020). Notably, the reserved demeanour observed in young DMD patients during the interviews suggests that their limited questioning may stem from shyness or affected cognition and behaviour, rather than from full understanding (Snow et al., 2013; Latimer et al., 2017; Ricotti et al., 2017). This emphasizes the importance of age-appropriate and easy-to-understand materials, which can be ensured through co-creation with patients and patient representatives (McDonald et al., 2024). Such efforts should be prioritized as new DMD treatments become available, given their critical role in supporting shared decision making between families and clinicians.

### 4.2 Willingness to use gene therapy

Driven by limited satisfaction with corticosteroid treatment, the study revealed that gene therapy is often seen as a “dream therapy,” and this is reflected in the strong willingness to use gene therapy, even in the context of CTs, by all participants but one patient-caregiver pair. In line with Landrum et al. (2019), patients and caregivers primarily focused on the possible benefits of gene therapy, rather than the efficacy-related uncertainties. This is especially true in experienced patients, who tend to downplay the side effects of long-term corticosteroid use as a result of adaptation over time. The spontaneous expressions of willingness towards gene therapy observed in this study may be driven by patients’ and their families’ hope, or desperate desire for better options, reflecting both elevated expectations and sometimes disregarding current uncertainties. Consistent with literature, caregivers considered even a few extra quality-of-life years to be meaningful and expressed hope for the emergence of other treatments that may be developed in the meantime (Landrum et al., 2019).

Heslop et al. showed an especially high willingness of parents of older children to participate in gene therapy CTs. In contrast with these findings in the UK, we observed a great willingness of caregivers of younger (six to 10 years old) patients to try gene therapy in CT setting (Heslop et al., 2021). This difference can be explained by the fact that Heslop et al. investigated caregivers’ opinions via a survey explicitly questioning willingness towards gene therapy in CTs, whereas in these interviews, the willingness to use gene therapy in CT setting was mentioned spontaneously. As muscle damage is irreversible, it is unsurprising that caregivers were willing to consider gene therapy in CT setting for their young children to prevent muscle degeneration in early stages of the disease. Similarly, Jaffé et al. confirmed that in the case of cystic fibrosis, the majority (91%) of parents would let their child be treated with gene therapy in a CT (Jaffé et al., 2006). Interestingly, the parents of the two youngest children did not spontaneously express any willingness to try gene therapy in CT setting. As these children are still able to walk and have not yet needed corticosteroid treatment, this lack of spontaneously mentioning willingness can be attributed to the fact that the parents were not yet faced with this scenario.

Apparent was the fear of parents of making “wrong” decisions for their children, a finding that resonates with results from a study of gene therapy administration in children with haemophilia by Khair et al., showing that parents of especially young children with haemophilia were afraid of making the wrong choice (Khair et al., 2021).

The presented cases demonstrated how patients and caregivers balance the potential benefits of gene therapy against the side effects and uncertainties. The perceived benefits of gene therapy, even if uncertain, outweighed its side effects, exemplifying the value placed on temporary stabilisation. In contrast, the ranking exercise revealed subtle variations in preferences between patients and caregivers; however, such variability can be non-systematic, as highlighted by a systematic review and meta-analysis underscoring the complexity of assessing pediatric health outcomes and the importance of incorporating both perspectives in clinical practice and research (Nafees et al., 2025). While it also highlighted that the route of administration is of lesser importance, in line with a previous study of Landrum et al. (2019). The highest scoring characteristics in the ranking exercise demonstrate strong alignment with the number of times that they are mentioned in the literature on treatment expectations of DMD patients and caregivers (Paquin et al., 2019; Bridges et al., 2019; Peay et al., 2014; Landrum et al., 2019; Peay et al., 2024; Hollin et al., 2024). For future clinical trial design, these findings highlight the importance of considering existing patient preference data, or, if such data is unavailable, conducting dedicated studies to collect them. This ensures that trial endpoints are aligned with what truly matters to both patients and caregivers.

### 4.3 Attribute and level development

Various studies highlight the diverse factors influencing patients’ and caregivers’ choices, as well as the preference heterogeneity observed within and between these groups (Hollin et al., 2024; Nafees et al., 2025; Crossnohere et al., 2022). Factors they value as identified in literature include the effect on respiratory infections, cough strength, muscle function and heart function (Paquin et al., 2019; Bridges et al., 2019; Peay et al., 2014; Landrum et al., 2019; Hollin et al., 2016). Additionally, priorities shift along the disease trajectory: younger patients tend to value potential muscle function improvements more, whereas older patients are more focused on stabilization (Landrum et al., 2019). Furthermore, lung function benefits are more highly valued by non-ambulatory patients and their caregivers compared to their ambulatory counterparts (Paquin et al., 2019).

Overall, the selected attributes align well with the top five characteristics that patients and caregivers consider most influential in their treatment choices. This study qualitatively identified the treatment outcomes most important to DMD patients and demonstrated the practical value of qualitative research in shaping the identification and development of attributes and levels. These findings support their integration into subsequent quantitative research, for which an additional pilot will ensure DMD patients understand the attribute descriptions. For regulatory decision making, this provides a foundation for aligning indication labels with the treatment preferences of patients and caregivers. These insights can also inform health technology assessment and reimbursement decisions by ensuring that value judgments and outcome measures reflect what patients and caregivers consider most important.

### 4.4 Strengths and limitations

This study has several strengths and limitations to consider when interpreting the findings. A key strength of this study was the active involvement of patient representatives in the study design, including the review of the interview guide. Their input enhanced the validity of the study and helped optimize the clarity and relevance of information and questions for the target population. Their presence in the multidisciplinary advisory board played a crucial role throughout the attribute and level development process. Their expertise and that of the other stakeholders ensured that the attributes, levels, and corresponding explanations were clinically meaningful, comprehensible, and relevant to DMD patients and caregivers. Additionally, we achieved a heterogeneous sample in our qualitative research, which helps capture a broad range of perspectives across different disease severities. The full interview transcripts contributed to ensure patient-friendly wording for the attribute descriptions was used.

One limitation is the potential selection bias of participants as most participants had previously taken part in CT recruitment or expressed curiosity and interest in gene therapy, which may indicate a greater openness toward new treatments and may have influenced their responses toward a greater willingness to consider novel treatments. While the distribution of disease severity among interviewed DMD patients was well-balanced, the caregivers in the study mainly cared for DMD patients at the same disease stage. This could have influenced their responses, as perspectives may vary across different stages of DMD. Lastly, future studies should aim to include patients who have undergone gene therapy as their perspectives would provide unique and valuable insights that complement those of patients and caregivers considering such treatment. Despite these limitations, this study provides valuable insights into DMD patient and caregiver preferences, supporting the development of clinically relevant and patient-centred attributes for future preference elicitation studies.

## 5 Conclusion

The semi-structured interviews revealed key treatment outcomes that significantly impact DMD patients and caregivers, highlighting their hope for novel treatments that meet their needs. Despite uncertainties regarding efficacy and safety, most participants expressed willingness to use gene therapy. Parents of younger children, aged six to ten, may even consider enrolling them in a gene therapy clinical trial, as the disease causes irreversible and progressive muscle damage. Muscle function, heart function, social life impact, and self-care activities were rated as important by both patients and caregivers, while caregivers additionally prioritized heart and lung function, and patients emphasized short-term side effects and cough strength. Caregivers hoped for muscle stabilization to support mobility and independence, while patients wished to maintain their current abilities, with some hoping for physical improvement and the discontinuation of corticosteroids as this restricts their growth. The attribute development process underscored the value of bottom-up and top-down approaches, with a multi-stakeholder advisory board ensuring clinically and patient-relevant attribute descriptions and levels through expert consultation and CT data.

## Data Availability

The original contributions presented in the study are included in the article/Supplementary Material, further inquiries can be directed to the corresponding author.

## References

[B1] Abiiro G. A. Leppert G. Mbera G. B. Robyn P. J. De Allegri M. (2014). Developing attributes and attribute-levels for a discrete choice experiment on micro health insurance in rural Malawi. BMC Health Serv. Res. 14, 235. 10.1186/1472-6963-14-235 24884920 PMC4032866

[B2] Aguti S. Malerba A. Zhou H. (2018). The progress of AAV-mediated gene therapy in neuromuscular disorders. Expert Opin. Biol. Ther. 18 (6), 681–693. 10.1080/14712598.2018.1479739 29781327

[B3] Aiyegbusi O. L. Macpherson K. Elston L. Myles S. Washington J. Sungum N. (2020). Patient and public perspectives on cell and gene therapies: a systematic review. Nat. Commun. 11 (1), 6265. 10.1038/s41467-020-20096-1 33293538 PMC7722871

[B4] Annexstad E. J. Lund-Petersen I. Rasmussen M. (2014). Duchenne muscular dystrophy. Tidsskr. Nor. Laegeforen 134 (14), 1361–1364. 10.4045/tidsskr.13.0836 25096430

[B5] Arora H. (2019). Duchenne muscular dystrophy: still an incurable disease. Neurol. India 67 (3), 717–723. 10.4103/0028-3886.263203 31347542

[B6] Barthélémy F. Wein N. (2018). Personalized gene and cell therapy for duchenne muscular dystrophy. Neuromuscul. Disord. 28 (10), 803–824. 10.1016/j.nmd.2018.06.009 30224293

[B7] Beytía Mde L. Vry J. Kirschner J. (2012). Drug treatment of Duchenne muscular dystrophy: available evidence and perspectives. Acta Myol. 31 (1), 4–8. 22655510 PMC3440798

[B8] Birnkrant D. J. Bushby K. Bann C. M. Alman B. A. Apkon S. D. Blackwell A. (2018a). Diagnosis and management of Duchenne muscular dystrophy, part 2: respiratory, cardiac, bone health, and orthopaedic management. Lancet Neurol. 17 (4), 347–361. 10.1016/S1474-4422(18)30025-5 29395990 PMC5889091

[B9] Birnkrant D. J. Bushby K. Bann C. M. Apkon S. D. Blackwell A. Brumbaugh D. (2018b). Diagnosis and management of Duchenne muscular dystrophy, part 1: diagnosis, and neuromuscular, rehabilitation, endocrine, and gastrointestinal and nutritional management. Lancet Neurol. 17 (3), 251–267. 10.1016/S1474-4422(18)30024-3 29395989 PMC5869704

[B10] Bridges J. F. P. Tsai J. H. Janssen E. Crossnohere N. L. Fischer R. Peay H. (2019). How do members of the duchenne and becker muscular dystrophy community perceive a discrete-choice experiment incorporating uncertain treatment benefit? An application of research as an event. Patient 12 (2), 247–257. 10.1007/s40271-018-0330-8 30259384

[B11] Chamberlain J. R. Chamberlain J. S. (2017). Progress toward gene therapy for duchenne muscular dystrophy. Mol. Ther. 25 (5), 1125–1131. 10.1016/j.ymthe.2017.02.019 28416280 PMC5417844

[B12] Chang N. C. Chevalier F. P. Rudnicki M. A. (2016). Satellite cells in muscular dystrophy - Lost in Polarity. Trends Mol. Med. 22 (6), 479–496. 10.1016/j.molmed.2016.04.002 27161598 PMC4885782

[B13] Chew L. D. Bradley K. A. Boyko E. J. (2004). Brief questions to identify patients with inadequate health literacy. Fam. Med. 36 (8), 588–594. 15343421

[B14] Coast J. Al-Janabi H. Sutton E. J. Horrocks S. A. Vosper A. J. Swancutt D. R. (2012). Using qualitative methods for attribute development for discrete choice experiments: issues and recommendations. Health Econ. 21 (6), 730–741. 10.1002/hec.1739 21557381

[B15] Consortium T. P. (2022). “PREFER recommendations - Why,” in When and how to assess and use patient preferences in medical product decision-making.

[B16] Crisafulli S. Sultana J. Fontana A. Salvo F. Messina S. Trifirò G. (2020). Global epidemiology of Duchenne muscular dystrophy: an updated systematic review and meta-analysis. Orphanet J. Rare Dis. 15 (1), 141. 10.1186/s13023-020-01430-8 32503598 PMC7275323

[B17] Crone M. Mah J. K. (2018). Current and emerging therapies for duchenne muscular dystrophy. Curr. Treat. Options Neurol. 20 (8), 31. 10.1007/s11940-018-0513-6 29936551

[B18] Crossnohere N. Fischer R. Vroom E. Furlong P. Bridges J.(2022). A Comparison of caregiver and patient preferences for treating duchenne muscular dystrophy. Patient - Patient-Centered Outcomes Res. 15, 577, 588. 10.1007/s40271-022-00574-y 35243571 PMC8894129

[B19] Davis J. Samuels E. Mullins L. (2015). Nutrition Considerations in duchenne muscular dystrophy. Nutr. Clin. Pract. 30 (4), 511–521. 10.1177/0884533615586202 25977513

[B20] Falzarano M. S. Scotton C. Passarelli C. Ferlini A. (2015). Duchenne muscular dystrophy: from diagnosis to therapy. Molecules 20 (10), 18168–18184. 10.3390/molecules201018168 26457695 PMC6332113

[B21] Fransen M. P. Van Schaik T. M. Twickler T. B. Essink-Bot M. L. (2011). Applicability of internationally available health literacy measures in The Netherlands. J. Health Commun. 16 (Suppl. 3), 134–149. 10.1080/10810730.2011.604383 21951248

[B22] Gale N. K. Heath G. Cameron E. Rashid S. Redwood S. (2013). Using the framework method for the analysis of qualitative data in multi-disciplinary health research. BMC Med. Res. Methodol. 13, 117. 10.1186/1471-2288-13-117 24047204 PMC3848812

[B23] Gärtner F. R. Portielje J. E. Langendam M. Hairwassers D. Agoritsas T. Gijsen B. (2019). Role of patient preferences in clinical practice guidelines: a multiple methods study using guidelines from oncology as a case. BMJ Open 9 (12), e032483. 10.1136/bmjopen-2019-032483 31811009 PMC6924854

[B24] George M. Apter A. J. (2004). Gaining insight into patients' beliefs using qualitative research methodologies. Curr. Opin. Allergy Clin. Immunol. 4 (3), 185–189. 10.1097/00130832-200406000-00008 15126939

[B25] Guglieri M. Bushby K. McDermott M. P. Hart K. A. Tawil R. Martens W. B. (2017). Developing standardized corticosteroid treatment for Duchenne muscular dystrophy. Contemp. Clin. Trials 58, 34–39. 10.1016/j.cct.2017.04.008 28450193 PMC6279424

[B26] Hauber B. Coulter J. (2020). Using the threshold technique to elicit patient preferences: an introduction to the method and an overview of existing Empirical Applications. Appl. Health Econ. Health Policy 18 (1), 31–46. 10.1007/s40258-019-00521-3 31541362

[B27] Heslop E. Turner C. Irvin A. Muntoni F. Straub V. Guglieri M. (2021). Gene therapy in Duchenne muscular dystrophy: identifying and preparing for the challenges ahead. Neuromuscul. Disord. 31 (1), 69–78. 10.1016/j.nmd.2020.10.001 33158687 PMC7564510

[B28] Hoffman E. P. (2020). Pharmacotherapy of duchenne muscular dystrophy. Handb. Exp. Pharmacol. 261, 25–37. 10.1007/164_2019_256 31375923

[B29] Hollin I. A.-O. Peay H. Fischer R. Janssen E. M. Bridges J. F. P. (2024). Engaging patients and caregivers in prioritizing symptoms impacting quality of life for Duchenne and Becker muscular dystrophy, 1573–2649. (Electronic)). 10.1007/s11136-018-1891-7 29804169

[B30] Hollin I. L. Caroline Y. Hanson C. Bridges J. F. P. Peay H. (2016). Developing a patient-centered benefit-risk survey: a community-engaged process. Value Health 19 (6), 751–757. 10.1016/j.jval.2016.02.014 27712702

[B31] Hollin I. L. Peay H. L. Apkon S. D. Bridges J. F. P. (2017). Patient-centered benefit-risk assessment in duchenne muscular dystrophy. Muscle Nerve 55 (5), 626–634. 10.1002/mus.25411 27649378

[B32] Hollin I. L. Craig B. M. Coast J. Beusterien K. Vass C. DiSantostefano R. (2020). Reporting Formative qualitative research to support the development of quantitative preference study protocols and corresponding survey Instruments: guidelines for authors and Reviewers. Guidel. Authors Rev. Patient 13 (1), 121–136. 10.1007/s40271-019-00401-x 31840215

[B33] Iannaccone S. T. Nanjiani Z. (2001). Duchenne muscular dystrophy. Curr. Treat. Options Neurol. 3 (2), 105–117. 10.1007/s11940-001-0045-2 11180747

[B34] Jaffé A. Prasad S. A. Larcher V. Hart S. (2006). Gene therapy for children with cystic fibrosis--who has the right to choose? J. Med. Ethics 32 (6), 361–364. 10.1136/jme.2005.012740 16731738 PMC2563368

[B35] Janssens R. Huys I. van Overbeeke E. Whichello C. Harding S. Kübler J. (2019). Opportunities and challenges for the inclusion of patient preferences in the medical product life cycle: a systematic review. BMC Med. Inf. Decis. Mak. 19 (1), 189. 10.1186/s12911-019-0875-z 31585538 PMC6778383

[B36] Jimenez-Moreno A. C. Pinto C. A. Levitan B. Whichello C. Dyer C. Van Overbeeke E. (2020). A study protocol for quantifying patient preferences in neuromuscular disorders: a case study of the IMI PREFER Project. Wellcome Open Res. 5, 253. 10.12688/wellcomeopenres.16116.1 34395923 PMC8356266

[B37] Ke Q. Zhao Z. Y. Mendell J. R. Baker M. Wiley V. Kwon J. M. (2019). Progress in treatment and newborn screening for Duchenne muscular dystrophy and spinal muscular atrophy. World J. Pediatr. 15 (3), 219–225. 10.1007/s12519-019-00242-6 30904991

[B38] Kerr C. Nixon A. Wild D. (2010). Assessing and demonstrating data saturation in qualitative inquiry supporting patient-reported outcomes research. Expert Rev. Pharmacoecon Outcomes Res. 10 (3), 269–281. 10.1586/erp.10.30 20545592

[B39] Khair K. Steadman L. Chaplin S. Holland M. Jenner K. Fletcher S. (2021). Parental perspectives on gene therapy for children with haemophilia: the Exigency study. Haemophilia 27 (1), 120–128. 10.1111/hae.14188 33216422

[B40] Kostek M. C. Gordon B. (2018). Exercise is an Adjuvant to Contemporary dystrophy treatments. Exerc Sport Sci. Rev. 46 (1), 34–41. 10.1249/JES.0000000000000131 28857889

[B41] Landrum P. H. Fischer R. Tzeng J. P. Hesterlee S. E. Morris C. Strong Martin A. (2019). Gene therapy as a potential therapeutic option for Duchenne muscular dystrophy: a qualitative preference study of patients and parents. PLoS One 14 (5), e0213649. 10.1371/journal.pone.0213649 31042754 PMC6493713

[B42] Latimer R. Street N. Conway K. C. James K. Cunniff C. Oleszek J. (2017). Secondary Conditions among males with duchenne or Becker muscular dystrophy. J. Child Neurology 32 (7), 663–670. 10.1177/0883073817701368 28393671 PMC5502756

[B43] Liu X. Liu M. Wu L. Liang D. (2018). Gene therapy for Hemophilia and duchenne muscular dystrophy in China. Hum. Gene Ther. 29 (2), 146–150. 10.1089/hum.2017.213 29366352

[B44] Mah J. K. (2016). Current and emerging treatment strategies for Duchenne muscular dystrophy. Neuropsychiatr. Dis. Treat. 12, 1795–1807. 10.2147/NDT.S93873 27524897 PMC4966503

[B45] Manzur A. Y. Kuntzer T. Pike M. Swan A. (2004). Glucocorticoid corticosteroids for Duchenne muscular dystrophy. Cochrane Database Syst. Rev. (2), Cd003725. 10.1002/14651858.CD003725.pub2 15106215

[B46] Matthews E. Brassington R. Kuntzer T. Jichi F. Manzur A. Y. (2016). Corticosteroids for the treatment of Duchenne muscular dystrophy. Cochrane Database Syst. Rev. 2016 (5), Cd003725. 10.1002/14651858.CD003725.pub4 27149418 PMC8580515

[B47] McDonald I. R. Blocker E. S. Weyman E. A. Smith N. Dwyer A. A.-O. (2024). What are the best practices for Co-creating patient-Facing educational materials? A Scoping review of the literature. 10.3390/healthcare11192615 PMC10572900 37830651

[B48] McDonald C. M. Han J. J. Mah J. K. Carter G. T. (2012). Corticosteroids and Duchenne muscular dystrophy: does earlier treatment really matter? Muscle Nerve 45 (6), 777–779. 10.1002/mus.23304 22581529

[B49] Messina S. Vita G. L. (2018). Clinical management of Duchenne muscular dystrophy: the state of the art. Neurol. Sci. 39 (11), 1837–1845. 10.1007/s10072-018-3555-3 30218397

[B50] Nafees Z. O'Neill S. Dimmer A. Guadagno E. Ferreira J. Mayo N. (2025). Child- and Proxy-reported differences in patient-reported outcome and experience measures in pediatric Surgery: systematic review and meta-analysis. J. Pediatr. Surg. 60 (5), 162172. 10.1016/j.jpedsurg.2025.162172 39865004

[B51] Okada T. Takeda S. (2013). Current challenges and future Directions in recombinant AAV-mediated gene therapy of duchenne muscular dystrophy. Pharm. (Basel) 6 (7), 813–836. 10.3390/ph6070813 24276316 PMC3816704

[B52] Paquin R. S. Fischer R. Mansfield C. Mange B. Beaverson K. Ganot A. (2019). Priorities when deciding on participation in early-phase gene therapy trials for Duchenne muscular dystrophy: a best-worst scaling experiment in caregivers and adult patients. Orphanet J. Rare Dis. 14 (1), 102. 10.1186/s13023-019-1069-6 31072340 PMC6509771

[B53] Peay H. A.-O. Fischer R. Mange B. Paquin R. S. Smith E. C. Sadosky A. (2024). Patients' and caregivers' maximum acceptable risk of death for non-curative gene therapy to treat Duchenne muscular dystrophy, 2324–9269. (Electronic)). 10.1002/mgg3.1664 PMC8172191 33755338

[B54] Peay H. L. Hollin I. Fischer R. Bridges J. F. (2014). A community-engaged approach to quantifying caregiver preferences for the benefits and risks of emerging therapies for Duchenne muscular dystrophy. Clin. Ther. 36 (5), 624–637. 10.1016/j.clinthera.2014.04.011 24852596

[B55] Peters K. Halcomb E. (2015). Interviews in qualitative research. Nurse Res. 22 (4), 6–7. 10.7748/nr.22.4.6.s2 25783145

[B56] Reinig A. M. Mirzaei S. Berlau D. J. (2017). Advances in the treatment of duchenne muscular dystrophy: new and emerging pharmacotherapies. Pharmacotherapy 37 (4), 492–499. 10.1002/phar.1909 28152217

[B57] Ricotti V. Mandy W. P. Scoto M. Pane M. Deconinck N. Messina S. (2017). Neurodevelopmental, emotional, and behavioural problems in Duchenne muscular dystrophy in relation to underlying dystrophin gene mutations, 1469–8749. 10.1111/dmcn.12922 26365034

[B58] Rydén A. Chen S. Flood E. Romero B. Grandy S. (2017). Discrete choice experiment attribute selection using a Multinational interview study: treatment Features important to patients with type 2 Diabetes Mellitus. Patient 10 (4), 475–487. 10.1007/s40271-017-0225-0 28315192

[B59] Settumba S. N. Shanahan M. Butler T. Schofield P. Lafferty L. Simpson P. (2019). Developing attributes and attribute-levels for a discrete-choice experiment: an example for Interventions of Impulsive Violent Offenders. Appl. Health Econ. Health Policy 17 (5), 683–705. 10.1007/s40258-019-00484-5 31161367

[B60] Shieh P. B. (2018). Emerging strategies in the treatment of duchenne muscular dystrophy. Neurotherapeutics 15 (4), 840–848. 10.1007/s13311-018-00687-z 30414046 PMC6277306

[B61] Shimizu-Motohashi Y. Miyatake S. Komaki H. Takeda S. Aoki Y. (2016). Recent advances in innovative therapeutic approaches for Duchenne muscular dystrophy: from discovery to clinical trials. Am. J. Transl. Res. 8 (6), 2471–2489. Available online at: www.ajtr.org/ISSN:1943-8141/AJTR0025 27398133 PMC4931144

[B62] Snow W. M. Anderson J. E. Jakobson L. S. (2013). Neuropsychological and neurobehavioral functioning in Duchenne muscular dystrophy: a review. Neurosci. and Biobehav. Rev. 37 (5), 743–752. 10.1016/j.neubiorev.2013.03.016 23545331

[B63] Soekhai V. Whichello C. Levitan B. Veldwijk J. Pinto C. A. Donkers B. (2019). Methods for exploring and eliciting patient preferences in the medical product lifecycle: a literature review. Drug Discov. Today 24 (7), 1324–1331. 10.1016/j.drudis.2019.05.001 31077814

[B64] Sun C. Serra C. Lee G. Wagner K. R. (2020). Stem cell-based therapies for Duchenne muscular dystrophy. Exp. Neurol. 323, 113086. 10.1016/j.expneurol.2019.113086 31639376 PMC6899334

[B65] Suthar R. Sankhyan N. (2018). Duchenne muscular dystrophy: a practice Update. Indian J. Pediatr. 85 (4), 276–281. 10.1007/s12098-017-2397-y 28653137

[B66] Thorne B. Takeya R. Vitelli F. Swanson X. (2018). Gene therapy. Adv. Biochem. Eng. Biotechnol. 165, 351–399. 10.1007/10_2016_53 28289769

[B67] Tong A. Sainsbury P. Craig J. (2007). Consolidated criteria for reporting qualitative research (COREQ): a 32-item checklist for interviews and focus groups. Int. J. Qual. Health Care 19 (6), 349–357. 10.1093/intqhc/mzm042 17872937

[B68] van Overbeeke E. Hauber B. Michelsen S. Goldman M. Simoens S. Huys I. (2021). Patient preferences to assess value IN gene therapies: protocol development for the PAVING study in Hemophilia. Front. Med. (Lausanne) 8, 595797. 10.3389/fmed.2021.595797 33768101 PMC7985056

[B69] van Overbeeke E. Whichello C. Janssens R. Veldwijk J. Cleemput I. Simoens S. (2019). Factors and situations influencing the value of patient preference studies along the medical product lifecycle: a literature review. Drug Discov. Today 24 (1), 57–68. 10.1016/j.drudis.2018.09.015 30266656

[B70] van Overbeeke E. Forrester V. Simoens S. Huys I. (2021a). Use of patient preferences in health technology assessment: perspectives of Canadian, Belgian and German HTA representatives. Belg. Ger. HTA Represent. Patient. 14 (1), 119–128. 10.1007/s40271-020-00449-0 32856278 PMC7794204

[B71] van Overbeeke E. Michelsen S. Hauber B. Peerlinck K. Hermans C. Lambert C. (2021b). Patient perspectives regarding gene therapy in haemophilia: interviews from the PAVING study. Haemophilia 27 (1), 129–136. 10.1111/hae.14190 33161616 PMC7894464

[B72] Verhaart I. E. Aartsma-Rus A. (2012). Gene therapy for Duchenne muscular dystrophy. Curr. Opin. Neurol. 25 (5), 588–596. 10.1097/WCO.0b013e328357b0be 22892952

[B73] Verhaart I. E. C. Aartsma-Rus A. (2019). Therapeutic developments for Duchenne muscular dystrophy. Nat. Rev. Neurol. 15 (7), 373–386. 10.1038/s41582-019-0203-3 31147635

[B74] Walter M. C. Reilich P. (2017). Recent developments in Duchenne muscular dystrophy: facts and numbers. J. Cachexia Sarcopenia Muscle 8, 681–685. Germany2017. 10.1002/jcsm.12245 29076660 PMC5659056

[B75] Yiu E. M. Kornberg A. J. (2015). Duchenne muscular dystrophy. J. Paediatr. Child. Health 51 (8), 759–764. 10.1111/jpc.12868 25752877

