# Supplementary material for: Preferences for gene therapy in Duchenne muscular dystrophy: insights from patient and caregiver interviews and attribute development
Source: Front Pharmacol. 2025 Sep 29;16:1662586. doi: 10.3389/fphar.2025.1662586 (PMC12515650; doi:10.3389/fphar.2025.1662586)
Supplement: Supplementary file 1 [file Supplementaryfile1.pdf]

Appendix I - Interview guide

Part A: Introduction

Number interview	
Date	
Name of interviewer	
Start time	
End time	

The information below will be discussed verbally with the interviewee, using visual support.

My name is the name of the interviewer (and my colleague is the name of the supervisor if present). The purpose of today's interview is to find out what patients think about a new, innovative therapy in research, called gene therapy, in the treatment of Duchenne muscular dystrophy. This research is part of Sophie Vermeire 's master's thesis. This research is being conducted in collaboration with various organizations, including hospitals and patient organizations.

Specifically, we will discuss the following topics over the next hour:

- We will talk about gene therapy, an innovative treatment that has not yet been approved in Europe and is therefore not yet available for the treatment of Duchenne muscular dystrophy.
- We will first check what you already know about gene therapy, after which we will provide you with information about it and check whether it is understandable.
- We will then ask your opinion about this therapy and ask why you would/would not use this therapy.
- We will also review with you some case studies of patients in clinical trials who have received this therapy and ask you how you would make a choice in this situation.
- We will ask you how you experience the uncertainties that still exist around gene therapy.

I want to thank you again for participating in this interview. Before we start, I want to make a few things clear:

- We are looking for your opinion and there are no wrong answers.
- If the patient is also present, we would like to hear the opinion of both the patient and the caregiver. Therefore, we will first ask the patient the question. After the patient has answered, the caregiver may add anything or give his/her own opinion. If the patient wishes, he/she may also consult with the caregiver before answering.
- This interview is completely confidential and will be processed in a non-identifiable manner.

- The interview will last approximately one hour.
- You are not obligated to answer any questions you do not wish to answer.
- You can stop the interview at any time without having to give a reason.
- If you did not understand a particular question or have any further questions, please feel free to ask.

Is the consent form filled in and signed? This must be in order before the interview can start. If not: do not continue with the interview until the consent form is signed.

In order to process the information from this interview and report it correctly, I would like to record this interview, audio only. This way I can transcribe the entire interview afterwards and process it correctly. Do you agree that I start the recording now? *Only start recording if you agree. If the participant does not agree and all questions about the recording have been answered, the interview will be stopped.*

Part B: Interview with Duchenne patient and/or caregiver

Introduction

If the introductory questionnaire has not yet been received by e-mail: could I ask you to e-mail me the questionnaire that was sent to you in advance? *When the questionnaire is received, it will be checked whether all questions have been answered. If there are still open questions in the questionnaire:* Would you like to answer the question regarding *'open question'* or would you rather leave it open? *If answer: answer is becoming added to the questionnaire.*

Introductory question:

- Why did you agree to participate in this study?

Gene therapy – information section

- Have you ever heard of gene therapy?
    - If yes, what do you already know about gene therapy?
    - If yes, how did you obtain this information?

- How do you rate your knowledge of gene therapy?
    - Very good
    - Good
    - Reasonable
    - Bad
    - Very bad

Now I am going to go over information with you about the disease, current treatment and the use of gene therapy in Duchenne muscular dystrophy. I will ask you each time if you have understood this information sufficiently and how I could possibly express this information more clearly. You may already know certain aspects of this information, but I would still like to receive your feedback.

Information about Duchenne muscular dystrophy

In patients with Duchenne muscular dystrophy, there is an **error in the gene** for the dystrophin protein. As a result, this **protein is not produced or is not produced properly.** Dystrophin protein works like a **shock absorber** and ensures muscle **strength.** Due to a deficiency of this protein, the body of patients with Duchenne muscular dystrophy can no longer repair damage to the muscles and the **muscles become increasingly weak.**

- Did you understand this information?
  - How could this information be expressed more clearly?

Information about the treatment of Duchenne muscular dystrophy

Today's treatments **cannot cure Duchenne muscular dystrophy.** The goal of treatment for Duchenne muscular dystrophy is to relieve **symptoms and support patients. Corticosteroids** are used to slow the decline and loss of muscle strength. This medication must be taken daily. Patients are also treated with physiotherapy and heart medications. At a certain age, they need a wheelchair to get around.

- Did you understand this information?
  - How could this information be expressed more clearly?

General information about gene therapy for Duchenne muscular dystrophy

Genetic disorders are caused by an error that occurs in our genetic material, or in other words an **error in one of our genes.** This error can occur **spontaneously** or can be **passed on via the mother**. By means of gene therapy, a therapy that is still under investigation, we try **to correct this error** so that the body once again contains the correct gene from which a certain protein is made. The goal of gene therapy for Duchenne muscular dystrophy is to deliver a **more correct version of the gene for the dystrophin protein into the body.** This gene that is given is not the full correct gene. Through gene therapy, the patient can produce **active dystrophin** himself, which will **slow down the progression** of the disease.

- Did you understand this information?
  - How could this information be expressed more clearly?

In gene therapy, a more correct version of the dystrophin **gene will be directly administered**. The target gene is always packaged in a "**vector** " that is responsible for delivering the gene to the muscle cells. For Duchenne muscular dystrophy, a **modified virus is** used as a vector because these have a good capacity to reach the muscle cells. Only the coat of the virus remains as a kind of **taxi**. The virus is modified in such a way that it is only able to bring the genes to the muscle cells, but is not able to make you ill. In the muscle cells, **the new gene is** added in addition to the already existing genetic material. The patient's own genetic material is not modified.

- Did you understand this information?
  - How could this information be expressed more clearly?

Practical information about the treatment of Duchenne muscular dystrophy with gene therapy

Gene therapy is administered **once through the vein** for 1 to 2 hours in the hospital. After this administration, only follow-up is necessary. After the vector has delivered the gene to the muscle cells, the muscle cells themselves start to produce a **partially working dystrophin protein.** The gene therapy treatment ensures that your body will produce active dystrophin. As a result, it is expected that **muscle strength** will stabilize, decrease less quickly or perhaps increase slightly. It is also expected that there **may** be an effect on the **heart and breathing.** In the first 2 weeks after the treatment, patients come for a check-up almost daily for blood sampling, then weekly for up to 3 months. Over time, check-ups become less frequent and from six months after the treatment, patients still come for a check-up every six months.

- Do you feel you understood the information well?
    - How could this information be expressed more clearly?

Approximately 30-80% of the population already has **antibodies** against the viral vectors used. This means that these people are currently not eligible for gene therapy because **the vector will be broken down by their body.** Due to the antibodies present, the vector cannot reach the muscle cells and the gene therapy will not be effective. When patients **without pre-existing antibodies participate in clinical studies, it is found that they develop antibodies against the vector** after administration of the gene therapy. This means that they can be successfully treated once with the same vector, but that this treatment with the same vector cannot be repeated. It is not yet known whether it is possible to treat patients again with a different type of vector to which the patient does not yet have antibodies if the gene therapy does not have a lasting effect.

- Do you feel you understood the information well?
    - How could this information be expressed more clearly?

Efficacy according to clinical studies for gene therapy in Duchenne muscular dystrophy

This therapy is still in clinical development and has not yet been approved by the European Commission. All data we cite here come from clinical studies. In these studies we see that after treatment with gene therapy there is a **stabilization** or **sometimes an increase** in dystrophin protein in a number of muscles. An improvement in muscle function was also observed in some patients. This therapy cannot undo damage that has already occurred, but it is expected that further damage can be prevented. The studies are still in an early stage. It is currently estimated that this therapy will have an **effect** for **about 10 years,** but there is **no certainty** about this at this time. In other words: **it is not yet known whether this therapy will continue to have a beneficial effect for life.**

- Did you understand this information?
    - How could this information be expressed more clearly?

Side effects of gene therapy in Duchenne muscular dystrophy.

After treatment with gene therapy, some patients experience mild side effects such as **nausea, vomiting, decreased appetite.** These always disappear after about 1 week. In only a few cases, more serious side effects can occur in the first two weeks after treatment. These include a reaction of the **immune system, or dehydration** due to persistent vomiting. These more serious side effects are **monitored as closely as possible** after treatment.

- Did you understand this information?

    - How could this information be expressed more clearly?

Gene therapy – patients - and caregivers opinion

- What is your opinion on treating Duchenne muscular dystrophy with gene therapy?
    - What additional information would you like to receive regarding gene therapy for Duchenne muscular dystrophy?
    - Through which channels would you like to receive this information?
- Would you be willing to have yourself treated with gene therapy/Would you be willing to have your child treated with gene therapy? Why or why not?
    - Can you indicate your willingness to use gene therapy on this scale:
        - ☐ Very willing
        - ☐ Willing
        - ☐ Neutral
        - ☐ Not willing
        - ☐ Absolutely not willing
    - Are there any other elements that could influence your choice that we have not already discussed in the information section?
    - What are the top three elements that influence your choice?
        - ☐ Place them in the table below
        - ☐ What would be meaningful changes for you?

- What is your view on the fact that it is not yet known how long gene therapy will be effective since there is no life-long data yet?
- How many years of proven efficacy do you need to have before you would consider gene therapy treatment?
- What is your view on the fact that we do not yet know what potential long-term side effects there are since there is no lifelong data yet?
- How many years of proven safety do you need before you would consider gene therapy treatment?
- Today, only patients without antibodies against the vector can be treated. What is your position on this?
- In addition, you can only be treated once with the same vector because you develop antibodies after the treatment. They are currently investigating whether it is possible to be treated a second time with a different vector. What is your position on this?
- In the table below, please indicate the **six elements** that you consider to be the most important in deciding whether gene therapy is the appropriate treatment for you/your child. Please then give them a score from 1 (most important) to 6 (least important).

Categories	**Elements**	*Definition*	***Patient score***	***Caregiver Score***
Nature of treatment	**Mechanism of action**	*The process by which a treatment has an effect (e.g. by delivering a gene to the muscle cells in the case of gene therapy)*		
Administration	**Method of administration**	*The route in which the treatment is administered to the patient (e.g., oral, intravenous, intramuscular)*		
	**Dosage frequency**	*How often the patient is treated within a specific period (e.g. single administration, daily,…)*		
Follow-up	**Frequency of follow-up**	*The number of times the patient must consult a doctor to monitor the effect of the treatment. (e.g. weekly, monthly, yearly)*		
	**Period of follow-up**	*How long or for what period follow-up is needed after treatment.*		
Advantages	**Effect on muscle function**	*The effect of the treatment on muscle strength and function (e.g. distance that can be covered, progression of strength loss)*		
	**Effect on lung function**	*The effect of treatment on lung function*		
	**Effect on the heart**	*Effect of treatment on the heart*		
	**Uncertainty about long-term efficacy**	*Uncertainty about how long the desired effects will be observed after treatment.*		
	**Effect on cough force**	*The effect of treatment on cough force.*		
	**The chance that corticosteroid use can be stopped**	*The possibility that corticosteroid use can be stopped after treatment.*		
Quality of life	**Impact on performing self-care activities, independence**	*Impact of treatment on the ability to perform activities independently.*		

	**Impact on the patient's social life**	*The effect of treatment on participation in social activities.*		
	**Impact on the caregiver's social life**	*The effect of treatment on participation in social activities.*		
Risks	**Risk of immune response**	*The risk that the body will develop antibodies against the vector used in the treatment.*		
	**Risk of experiencing side effects in the short term**	*The risk of experiencing side effects such as nausea, decreased appetite, vomiting, etc. after treatment.*		
	**Uncertainty regarding long-term risks**	*The degree of uncertainty regarding the adverse events that may occur following administration of the treatment (uncertainty may exist due to the limited time patients were followed up after administration or due to a limited number of patients treated with the treatment)*		
Spontaneously mentioned elements				

- Which elements on this list do you consider absolutely not important when making a decision about gene therapy treatment?

Case

I am going to walk you through two hypothetical examples based on publicly published clinical studies with Duchenne muscular dystrophy patients. I will ask you afterwards whether you would prefer gene therapy or supportive therapy in that case:

Example 1: An 8-year-old patient has been diagnosed with Duchenne muscular dystrophy and does not yet require a wheelchair. The patient can choose between the following two treatments:

**Symptomatic treatment**	**Gene therapy**
**Administration:** Take corticosteroids orally daily.	**Administration:** After several months of corticosteroid treatment, a single administration of a vector with a more correct dystrophin gene is given via the

	vein. This more correct gene is then added next to the genetic material in the muscle cells.
**Effect:** Despite treatment, there is still a slower but further increase in muscle weakness.	**Effect:** A dystrophin concentration is reached at which **no further progression** of muscle weakness is observed. An **increase in muscle strength may also be** observed. However, there is no certainty yet as to how long the treatment will be effective, as there are no lifelong data available.
**Possible side effects:**<br><br>Side effects of long-term use of corticosteroids such as weight gain, osteoporosis, …	**Possible side effects:**<br><br>- Nausea, vomiting, decreased appetite in the first week after administration<br>- Development of immunity (antibodies) against the vector used<br>- There is still some uncertainty about potential long-term side effects because no lifelong data are available yet

- Did you understand this example?
- Would you prefer gene therapy or symptomatic therapy in this case?
    - Why?

Example 2: An 8-year-old patient has been diagnosed with Duchenne muscular dystrophy and does not yet require a wheelchair. The patient can choose between the following two treatments:

**Symptomatic treatment**	**Gene therapy**
**Administration:** Take corticosteroids orally daily.	**Administration:** After several months of corticosteroid treatment, a single administration of a vector with a more correct dystrophin gene is given via the vein. This more correct gene is then added next to the genetic material in the muscle cells.
**Effect:** Despite treatment, there is still a slower but further increase in muscle weakness.	**Effect:** A dystrophin concentration is reached where there is a temporary stabilization of muscle strength, followed by a slow progression. However, there is no certainty yet how long the treatment will be effective since there are no lifelong data available.

**Possible side effects:**<br>Side effects of long-term use of corticosteroids such as weight gain, osteoporosis, …	**Possible side effects:**<br>- Nausea, vomiting, and decreased appetite in the first week after administration<br>- Development of immunity (antibodies) against the vector used<br>-There is still some uncertainty about potential long-term side effects because no lifelong data are available yet

- Did you understand this example?
- Would you prefer gene therapy or symptomatic therapy in this case?
  - Why?
- If both answers to the previous two questions were the same: why did you answer gene therapy/preventive therapy to both examples?
- If the answers to the previous two questions were different: why did you give a different answer to the previous two examples?

Completion of the interview

- We've gone over all the questions from the interview. Is there anything else you'd like to share with me that I haven't asked yet?
- Do you have any questions for me?
- What did you think of the interview?

Thank you very much for participating in this interview. Please do not hesitate to contact me if you have any questions.

Appendix II – Systematic literature searchLiterature search of clinical trials

Following search terms (Table A 1) were used to identify literature on gene therapy in Duchenne muscular dystrophy in PubMed. Additionally, the search terms "Duchenne Muscular Dystrophy" and "Gene therapy" were used to search in clinicaltrials.gov (Figure A 1).

Table A 1 Search terms used to identify literature on clinical trials with gene therapy in DMD in PubMed.

Concept	MeSH	Free text (title and abstract)
Gene therapy	"Genetic Therapy"	"gene therapy", "gene therapies", "gene-therapy", "genetic therapy", "genetic therapies", "AAV"
Duchenne muscular dystrophy	"Muscular Dystrophy, Duchenne"	"Duchenne muscular dystrophy", "Duchenne's muscular dystrophy", "Duchenne's disease", "Duchenne type muscular dystrophy", "Duchenne"
Clinical trials	"Clinical Trials as Topic"	"clinical trial", "clinical trials", "clinical study", "clinical studies"

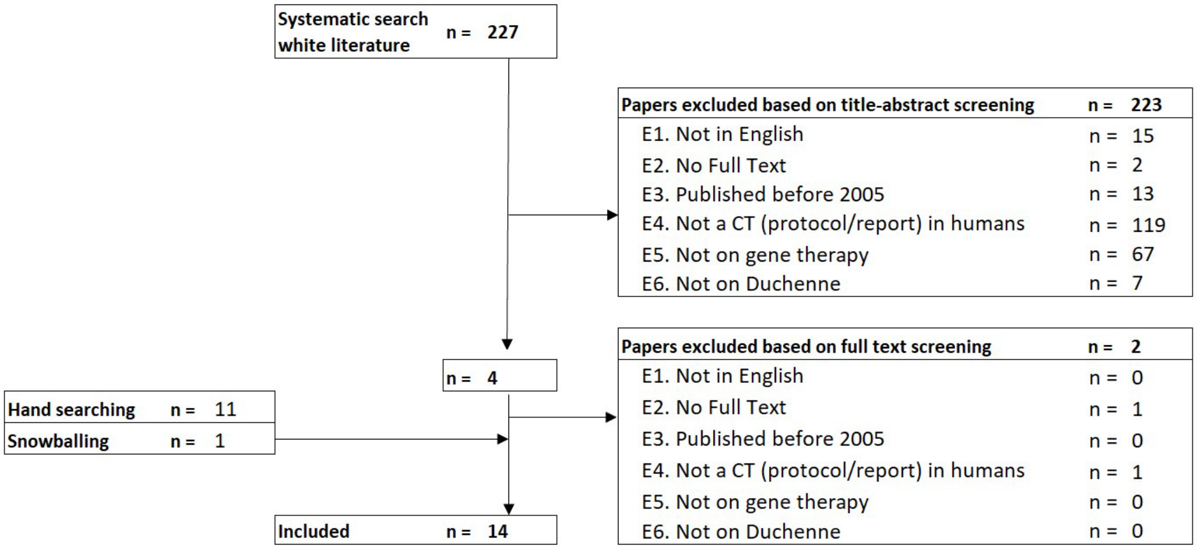

Figure A 1 Flow of the systematic literature search on clinical trials with gene therapy in DMD. Results of the initial search and the selection process of studies based on title-abstract screening, full-text screening, hand searching and snowballing. CT clinical trial

B) Literature search of patient preferences in DMD

Table A 2 Search terms used to identify literature on patient preferences in PubMed.

Concept	MeSH	Free text (title and abstract)
Patient preferences	"Patient Preference"	"patients preference", "patients preferences", "preference of patients", "preferences of patients", "patient preference", "patient preferences", "preference of a patient", "preference of the patient", "preferences of a patient", "preferences of the patient"
Duchenne	muscular dystrophy	"Muscular Dystrophy, Duchenne"

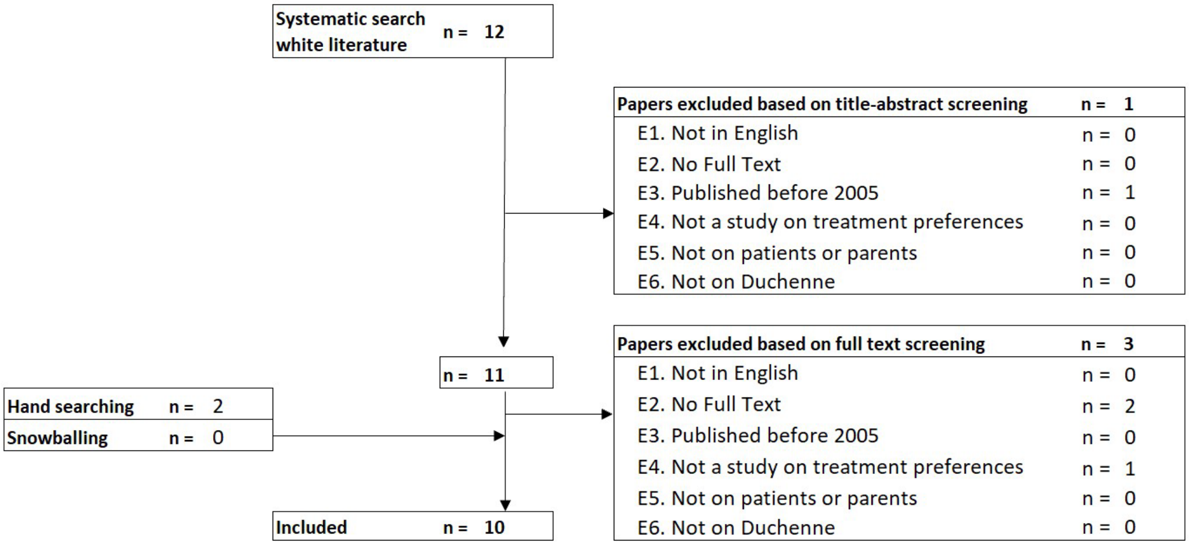

Figure A 2 Flow of the systematic literature search on patient preferences in DMD. Results of the initial search and the selection process of studies based on title-abstract screening, full-text screening, hand searching and snowballing.

Appendix III – Survey (Dutch)

A) Patiënt

Deze vragenlijst heeft als doel een algemeen beeld te schetsen van de interview-deelnemers. Op die manier kunnen de onderzoekers nagaan of de antwoorden die worden gegeven tijdens het interview een mogelijke link vertonen met de kenmerken die we in deze vragenlijst observeren. De antwoorden op deze vragenlijst zullen worden gepseudonymiseerd. Dit betekent dat een code wordt gegeven aan de verkregen informatie en dat alle informatie die u identificeerbaar zou maken wordt verwijderd uit de gegevens die gebruikt zullen worden voor analyse en rapportering van resultaten in rapporten en wetenschappelijke publicaties. Indien u het antwoord op een vraag niet weet of indien u liever niet antwoordt op een vraag kan u deze steeds open laten.

Leeftijd : ___________________

Waar woont u ?

□ Vlaanderen

□ Wallonië

□ Brussel Leeftijd bij diagnose:

Bent u in het verleden reeds met corticosteroïden behandeld ?

□ Ja

□ Neen

Wordt u op dit ogenblik met corticosteroïden behandeld ?

□ Ja

□ Neen

Hoe goed kan u nog stappen ?

□ Ik kan nog heel goed stappen

□ Verre afstanden en de trap doen zijn moeilijk

□ Ik kan enkel binnenshuis stappen

□ Ik kan enkele stapjes zetten (al dan niet met ondersteuning)

□ Ik kan niet meer stappen

Heeft u in het verleden reeds deelgenomen aan de rekrutering van een klinische studie?

□ Ja

□ Neen

Hoe tevreden bent u met uw huidige behandeling?

□ Zeer tevreden

□ Tevreden

□ Neutraal

□ Ontevreden

□ Zeer ontevreden

Hoe vaak helpt iemand u bij het lezen van informatiemateriaal van het ziekenhuis?

□ Altijd

□ Vaak

□ Soms

□ Zelden

□ Nooit

Hoe vaak heeft u problemen met het begrijpen van uw medische situatie doordat u moeite heeft met de schriftelijke informatie?

□ Altijd

□ Vaak

□ Soms

□ Zelden

□ Nooit

Hoe zeker bent u van uzelf als u zelf medische formulieren invult ?

□ Heel erg

□ Behoorlijk

□ Enigszins

□ Een klein beetje

□ Helemaal niet

Hartelijk dank voor het invullen van deze vragenlijst.

B) Verzorger

Deze vragenlijst heeft als doel een algemeen beeld te schetsen van de interview-deelnemers. Op die manier kunnen de onderzoekers nagaan of de antwoorden die worden gegeven tijdens het interview een mogelijke link vertonen met de kenmerken die we in deze vragenlijst observeren. De antwoorden op deze vragenlijst zullen worden gepseudonymiseerd. Dit betekent dat een code wordt gegeven aan de verkregen informatie en dat alle informatie die u identificeerbaar zou maken wordt verwijderd uit de gegevens die gebruikt zullen worden voor analyse en rapportering van resultaten in rapporten en wetenschappelijke publicaties. Indien u het antwoord op een vraag niet weet of indien u liever niet antwoordt op een vraag kan u deze steeds open laten.

Wat is uw relatie tot de patiënt?

□ Moeder

□ Vader

□ Wettelijke voogd

□ Naast familielid (+18j)

□ Volwassene dicht bij het gezin die in hetzelfde huis woont

□ Anders : ___________________

Uw leeftijd : ___________________

Uw geslacht :

□ Man

□ Vrouw

□ Anders

□ Ik geef hier liever geen antwoord op

Waar woont u?

□ Vlaanderen

□ Wallonië

□ Brussel

Indien u de moeder bent van de patiënt, bent u draagster van Duchenne spierdystrofie?

□ Ja

□ Neen

Leeftijd van de patiënt : ___________________

Leeftijd van de patiënt bij diagnose: ___________________

Hoe goed kan de patiënt nog stappen?

□ De patiënt kan nog heel goed stappen

□ Verre afstanden en de trap doen zijn moeilijk

□ De patiënt kan enkel binnenshuis stappen

□ De patiënt kan enkele stapjes zetten (al dan niet met ondersteuning)

□ De patiënt kan niet meer stappen

Is de patiënt in het verleden reeds met corticosteroïden behandeld ?

□ Ja

□ Neen

Wordt de patiënt op dit ogenblik met corticosteroïden behandeld?

□ Ja

□ Neen

Heeft de patiënt in het verleden reeds deelgenomen aan de rekrutering van een klinische studie?

□ Ja

□ Neen

Hoe tevreden bent u met de huidige behandeling van de patiënt?

□ Zeer tevreden

□ Tevreden

□ Neutraal

□ Ontevreden

□ Zeer ontevreden

Hoe vaak helpt iemand u bij het lezen van informatiemateriaal van het ziekenhuis?

□ Altijd

□ Vaak

□ Soms

□ Zelden

□ Nooit

Hoe vaak heeft u problemen met het begrijpen van uw medische situatie doordat u moeite heeft met de schriftelijke informatie?

□ Altijd

□ Vaak

□ Soms

□ Zelden

□ Nooit

Hoe zeker bent u van uzelf als u zelf medische formulieren invult ?

□ Heel erg
□ Behoorlijk
□ Enigszins
□ Een klein beetje
□ Helemaal niet

Hartelijk dank voor het invullen van deze vragenlijst.

Appendix IV – Saturation table

Code	Interview											
	**1**	**2**	**3**	**4**	**5**	**6**	**7**	**8**	**9**	**10**	**11**	**12**
**Information needs**												
Motivation to participate in the interview	X											
Awareness	X											
Previous information	X											
Source previous information	X											
Comprehension of disease	X											
Comprehension of treatment	X											
Comprehension of GT in general	X											
Comprehension of GT practical	X											
Comprehension of GT efficacy	X											
Comprehension of GT side effects	X											
Additional information	X											
Source additional information	X											
**Use of GT**												
Opinion GT	X											
Willingness to use GT	X											
Willing to participate in a clinical trial			X									
Top 3 influencing elements	X											

Additional influencing elements	X											
Not influencing elements	X											
Reason to use GT	X											
Meaningful change		X										
Reason to refrain	X											
Uncertainty efficacy	X											
Uncertainty safety	X											
Required years of evidence efficacy	X											
Required years of evidence safety	X											
Pre-existing antibodies	X											
Second-time vector	X											
**Cases**												
Comprehension case 1	X											
Choice treatment case 1	X											
Comprehension case 2	X											
Choice treatment case 2	X											
Compare choices	X											
**General**												
Opinion interview	X											

GT gene therapy; X when code was first applied

Appendix V – Individual attribute scores ranking exercise

Individual scores were summed and the five highest scores were marked in bold.

A) Patient

Patients	1	2	3	4	5	6	7	TOTAL SCORE
**Predefined attributes**								
Mechanism of action								0
Route of administration						2		2
Dose frequency								0
Frequency of monitoring								0
Period of monitoring		2						2
Effect on muscle function	6	6	2		4	6	6	**30**
Effect on lung function	3						3	6
Effect on the heart	4	5	3		5	5	4	**26**
Uncertainty long-term efficacy								0
Effect on cough strength	2	4		4				**10**
The probability that corticosteroid can be stopped	5				1		1	7
Impact on self-care activities, independence			4	3	3		5	**15**
Impact on patient's social life			6	2		1	2	**11**
Impact on caregiver's social life			5					5
Risk of immune response				1		3		4
Risk of short-term side effects		1	1	6	2			**10**
Uncertainty long-term risks		3		5				8
**Spontaneously mentioned attributes**								
Climb stairs	1							1
Treating physician's opinion on gene therapy					1			1
Information on the study						4		4

B) Caregiver

**Caregivers**	**1**	**2**	**3**	**4**	**5**	**6**	**7**	**8**	**9**	**10**	**11**	**TOTAL SCORE**
**Predefined attributes**												
Mechanism of action		1										1
Route of administration								2				2
Dose frequency			3									3
Frequency of monitoring												0
Period of monitoring												0
Effect on muscle function	6	6	4	4	6	4		4	6		6	**46**
Effect on lung function	3.5	4	5.5	1	4	5	1		5			**29**
Effect on the heart	3.5	3	5.5	5	5	6	2		4			**34**
Uncertainty long-term efficacy											3	3
Effect on cough strength						2						2
The probability that corticosteroid can be stopped		2		3		1	3		1			10
Impact on self-care activities, independence	1	5			3	3		6	3	5	5	**31**
Impact on patient's social life					1		5	1	2	4	2	**15**
Impact on caregiver's social life												0
Risk of immune response			1	6			4					11
Risk of short-term side effects	3											3
Uncertainty long-term risks			2	2	2			3		1	4	14
**Spontaneously mentioned attributes**												
The only chance for treatment for rare mutations	2											2
Negative experience study							6					6
Patient being well treated, treated as a patient and not a number								5		2		7
Patient's health										6		6
Honest and complete information on the treatment										3		3
Treats the cause											1	1

Appendix VI – Cases (English)

A) Case 1:

An eight-year-old patient is diagnosed with Duchenne muscular dystrophy and does not need a wheelchair. The patient can choose between the following two treatments:

Symptomatic treatment	Gene therapy
**Administration**: daily oral intake of corticosteroids	**Administration**: After several months of corticosteroid treatment, a one-time administration of a vector containing a more correct dystrophin gene via the vein follows. This more correct gene is subsequently added next to the genetic material in the muscle cells.
**Effect**: Despite treatment, there is still a slower, but further increase in muscle weakness.	**Effect**: A dystrophin concentration is reached at which no further progression of muscle weakness is observed. An increase in muscle strength may also be observed. However, there is no certainty yet how long the treatment will be effective as no lifelong data are yet available.
**Possible side effects**:<br><br>Side effects due to long-term use of corticosteroids such as weight gain, osteoporosis,...	**Possible side effects**:<br><br>- Nausea, vomiting, decreased appetite in the first week after administration<br>- Development of immunity (antibodies) against the vector used<br>- There is still some uncertainty about potential long-term side effects due to the lack of lifelong data on these.

B) Case 2:

An eight-year-old patient is diagnosed with Duchenne muscular dystrophy and does not need a wheelchair. The patient can choose between the following two treatments:

**Symptomatic treatment**	**Gene therapy**
**Administration**: daily oral intake of corticosteroids	**Administration**: After several months of corticosteroid treatment, a one-time administration of a vector containing a more correct dystrophin gene via the vein follows. This more correct gene is subsequently added next to the genetic material in the muscle cells
**Effect**: Despite treatment, there is still a slower, but further increase in muscle weakness.	**Effect**: A dystrophin concentration is reached at which there is a temporary stabilization of muscle strength, followed by a slow progression. However, there is no certainty on how long the treatment will be effective as lifetime data are not yet available.
**Possible side effects**:<br><br>Side effects due to long-term use of corticosteroids such as weight gain, osteoporosis,...	**Possible side effects**:<br><br>- Nausea, vomiting, decreased appetite in the first week after administration<br>- Development of immunity (antibodies) against the vector used<br>- There is still some uncertainty about potential long-term side effects due to the lack of lifelong data on these.

Appendix VII – Coding tree

1. Information needs

- 1.1. Motivation to participate in the interview
- 1.2. Awareness
- 1.3. Previous information gene therapy
  - 1.3.1. Source previous information
- 1.4. Comprehension of disease
- 1.5. Comprehension of treatment
- 1.6. Comprehension of gene therapy in general
- 1.7. Comprehension of gene therapy practical
- 1.8. Comprehension of gene therapy efficacy
- 1.9. Comprehension of gene therapy side effects
- 1.10. Additional information
  - 1.10.1. Source additional information

2. Use of gene therapy

- 2.1. Opinion gene therapy
- 2.2. Willingness to use gene therapy
  - 2.2.1. Willing to participate in a clinical trial
  - 2.2.2. Top 3 influencing elements
  - 2.2.3. Additional influencing elements
  - 2.2.4. Not influencing elements
- 2.3. Reason to use gene therapy
  - 2.3.1. Meaningful change
- 2.4. Reason to refrain
- 2.5. Uncertainties
  - 2.5.1. Uncertainty efficacy
  - 2.5.2. Uncertainty safety
- 2.6. Required years of evidence
  - 2.6.1. Required years of evidence efficacy
  - 2.6.2. Required years of evidence safety
- 2.7. Pre-existing antibodies
- 2.8. Second-time vector

3. Cases

3.1. Comprehension case 1

3.2. Choice treatment case 1

3.3. Comprehension case 2

3.4. Choice treatment case 2

3.5. Compare choices

4. General

4.1. Opinion interview

Appendix VIII – The COREQ checklist

No.	Questions	Response
**Domain 1: Research team and reflexivity**		
Personal Characteristics		
1	Which author/s conducted the interview?	SV conducted all interviews. RJ was present at the first 2 interviews
2	What were the researcher’s credentials?	SV holds a certificate of passing successfully the bridging year from biomedical laboratory technician to Biomedical Sciences and RJ holds an MSc in Biomedical Sciences
3	What was their occupation at the time of the study?	SV is completing her master’s in Biomedical Sciences, minor in management and communication.<br><br>RJ is completing her PhD in patient preference research at KU Leuven
4	Was the researcher male or female?	female
5	What experience or training did the researcher have?	SV followed training in completing qualitative interviews.<br><br>RJ had previous experience in completing qualitative interviews and has provided feedback after supervising the first two interviews
Relationship with participants		
6	Was a relationship established prior to study commencement?	No, participants were only contacted by mail or phone to agree on a time for the interview.
7	What did the participants know about the researcher?	The participants were informed on the goal of the research and the future use of the data.
8	What characteristics were reported about the interviewer/facilitator?	The fact that SV was studying Biomedical Sciences and that the research was part of her masters’ thesis.
**Domain 2: Study design**		
Theoretical framework		
9	What methodological orientation was stated to underpin the study?	Qualitative research evaluated through framework method analysis
Participant selection		

10	How were participants selected?	Individuals who met the inclusion criteria were contacted
11	How were participants approached?	Participants recruited via UZ Leuven were contacted by the clinical trial assistant of their physician. Caregivers from UCL Saint-Luc were contacted by the physician herself. Other participants were approached via the patient organisation (Duchenne Parent Project)
12	How many participants were in the study?	A total of 18 participants (7 patients and 11 caregivers) were interviewed
13	How many people refused to participate or dropped out? Reasons?	All participants that communicated to be willing to participate, were interviewed
Setting		
14	Where was the data collected?	Due to the Corona pandemic, interviews were organised online via a Skype or Zoom meeting. Participants and SV were each at home.
15	Was anyone else present besides the participants and researchers?	No, the only participant(s) and researcher SV were present (First two interviews RJ was also present)
16	What are the important characteristics of the sample?	See **Error! Reference source not found.**
Data collection		
17	Were questions, prompts, guides provided by the authors? Was it pilot-tested?	Yes, an interview guide was prepared and validated through input from a paediatric neurologist, neuropsychologist and members of the local patient organisation (parents). Two pilot interviews with a caregiver took place, one in French and one in Dutch.
18	Were repeat interviews carried out? If yes, how many?	No
19	Did the research use audio or visual recording to collect the data?	Yes, interviews were audio-recorded in double. For one interview the audio-recording was of too bad quality to be used.
20	Were field notes made during and/or after the interview?	Field notes were taken during the interview. After the interview audio recordings were fully transcribed.
21	What was the duration of the interviews?	45 minutes to 1 hour. Only one interview (with patient and caregiver) took 1,5 hours.
22	Was data saturation discussed?	Yes, a saturation table was made
23	Were transcripts returned to participants for comment and/or correction?	No

**Domain 3: analysis and findings**		
Data analysis		
24	How many data coders coded the data?	Two transcripts were independently coded by SV and EDS. The remaining coding was done by SV.
25	Did the authors provide a description of the coding tree?	No
26	Were themes identified in advance or derived from the data?	Both deductive and inductive codes were used.
27	What software, if applicable, was used to manage the data?	NVivo
28	Did participants provide feedback on the findings?	No
Reporting		
29	Were participant quotations presented to illustrate the themes / findings? Was each quotation identified?	Yes
30	Was there consistency between the data presented and the findings?	Yes
31	Were major themes clearly presented in the findings?	Yes
32	Is there a description of diverse cases or discussion of minor themes?	Yes

Appendix IX – Top three attributes

A) Top three attributes influencing treatment choice for individual patients

Patient	1	2	3	4	5	6	7
Walking	x	x					
Climb stairs	x						
Less short of breath	x						
Stop corticoids		x					
Prevent wheelchair		x					
Duration of the effect			x				
Physicians pay attention to the patient's opinion			x			x	
Side effects			x			x	
Stabilisation strength				x			x
Recover strength for respiration				x			
Recover strength in the arms				x			
Risks					x		
Improvement muscle strength					x		
Treating physician's opinion on gene therapy					x		
Information on the study						x	

Patient 7 could only mention one attribute.

B) Top three attributes influencing treatment choice for individual caregivers

Caregiver	1	2	3	4	5	6	7	8	9	10	11
Seeing positive effects in other patients	x	x									
Side effects	x					x	x				
The only chance for treatment for rare mutations	x										
Positive results in test persons (black on white)		x									
Patient’s willingness		x				x					
Time invested in the treatment			x								
Treats the cause of the disease			x								x
Only treatment			x								
Walk as long as possible				x							
Stabilisation muscle strength				x							
Live longer				x							
Prevent the need for a wheelchair					x						
Stop corticoids					x						
Living a normal life					x						
Not being able to participate in other studies						x	x				
Negative experience study							x				
Patient treated as a patient and not a number								x		x	
Distance to administration location								x			
Uncertainty side effects								x			
Being effective									x		
Not mutation-specific									x		
Single administration									x		x
Patient’s health										x	
Honest and complete information on the treatment										x	
Uncertainties											x

Appendix X – Table: List of unique identified characteristics (n=48) from literature (n=30) and from the semi-structured interviews (n=26)

	Characteristic description	References from literature	Mentioned during the interviews
1	Effect on muscle function	(57-61)	P - C
2	Effect on heart function	(57, 60, 62)	P - C
3	Effect on lung function	(57, 60, 62)	P - C
4	Uncertainty about the long-term risks		P - C
5	Probability that corticosteroid can be stopped		P - C
**6**	Impact on self-care activities, independence		P - C
7	Period of monitoring		P
**8**	Climbing stairs		P
9	Uncertainty of long-term efficacy		C
10	Dose frequency		C
11	Only chance for a treatment for rare mutations		C
12	Patient's health		C
13	Uncertainty about benefits	(57, 60, 61)	
14	Uncertainty about the duration of benefits	(57, 60)	
15	Ineligibility for most future clinical trials	(57, 60)	
16	Lifespan gain	(59)	
17	Knowledge about the drug	(59)	
18	Effect on cough strength▲	(38, 62)	P - C
19	Risk of immune response▲	(60)	P - C
20	Route and frequency of administration§	(60)	P - C
21	Risk of short-term side effects▲		P - C
22	Impact on patient's social life*§		P - C
23	Impact on caregiver's social life *°		P
24	Information on the study*		P
25	Treating physician's opinion on gene therapy°		P
26	Treats the cause§		C
27	Mechanism of action§		C
28	Patient being well treated, treated as a patient and not a number*§		C
29	Negative study experience *§		C
30	Honest and complete information on the treatment§		C
31	Reduction of lung infections▲	(38, 62)	
32	Risk of death▲	(57, 60)	
33	Risk of fractures▲	(58, 62)	
34	Risk of bleeds▲	(59)	
35	Risk of heart arrythmia▲	(59)	
36	Risk of long hospitalization▲	(57)	
37	Risk of kidney damage▲	(58)	
38	Additional blood draws▲	(38)	
39	Additional muscle biopsies▲	(57)	
40	Nausea▲	(59)	
41	Non-healthy weight▲	(62)	
42	Risk of diarrhoea▲	(38)	
43	Poor attention span*	(62)	
44	Frequent waking at night*	(62)	
45	Depression*▲	(62)	
46	Headaches*	(62)	
47	Constipation▲	(62)	
48	Feeling tired*	(62)	

Mentioned by patients (P) or caregivers (C). Characteristics not meeting the selection requirements: *Not treatment-related; §*not quantifiable;* °*Not patient-centered:* ▲*Overlapping characteristic*
